# A Low Dose of Dietary Resveratrol Partially Mimics Caloric Restriction and Retards Aging Parameters in Mice

**DOI:** 10.1371/journal.pone.0002264

**Published:** 2008-06-04

**Authors:** Jamie L. Barger, Tsuyoshi Kayo, James M. Vann, Edward B. Arias, Jelai Wang, Timothy A. Hacker, Ying Wang, Daniel Raederstorff, Jason D. Morrow, Christiaan Leeuwenburgh, David B. Allison, Kurt W. Saupe, Gregory D. Cartee, Richard Weindruch, Tomas A. Prolla

**Affiliations:** 1 LifeGen Technologies, LLC, Madison, Wisconsin, United States of America; 2 Department of Medical Genetics, University of Wisconsin, Madison, Wisconsin, United States of America; 3 Department of Genetics, University of Wisconsin, Madison, Wisconsin, United States of America; 4 Division of Kinesiology, University of Michigan, Ann Arbor, Michigan, United States of America; 5 Section on Statistical Genetics, Department of Biostatistics, University of Alabama at Birmingham, Birmingham, Alabama, United States of America; 6 Department of Medicine, University of Wisconsin-Madison, Madison, Wisconsin, United States of America; 7 R&D Human Nutrition and Health, DSM Nutritional Products Ltd., Basel, Switzerland; 8 Department of Pharmacology, Vanderbilt University School of Medicine, Nashville, Tennessee, United States of America; 9 Department of Medicine, Vanderbilt University School of Medicine, Nashville, Tennessee, United States of America; 10 Department of Aging and Geriatrics and College of Medicine, University of Florida, Gainesville, Florida, United States of America; 11 Section on Statistical Genetics, Department of Biostatistics and Clinical Nutrition Research Center, University of Alabama at Birmingham, Birmingham, Alabama, United States of America; 12 Department of Medicine and Veterans Administration Hospital, University of Wisconsin-Madison, Madison, Wisconsin, United States of America; AgroParisTech, France

## Abstract

Resveratrol in high doses has been shown to extend lifespan in some studies in invertebrates and to prevent early mortality in mice fed a high-fat diet. We fed mice from middle age (14-months) to old age (30-months) either a control diet, a low dose of resveratrol (4.9 mg kg^−1^ day^−1^), or a calorie restricted (CR) diet and examined genome-wide transcriptional profiles. We report a striking transcriptional overlap of CR and resveratrol in heart, skeletal muscle and brain. Both dietary interventions inhibit gene expression profiles associated with cardiac and skeletal muscle aging, and prevent age-related cardiac dysfunction. Dietary resveratrol also mimics the effects of CR in insulin mediated glucose uptake in muscle. Gene expression profiling suggests that both CR and resveratrol may retard some aspects of aging through alterations in chromatin structure and transcription. Resveratrol, at doses that can be readily achieved in humans, fulfills the definition of a dietary compound that mimics some aspects of CR.

## Introduction

Caloric restriction (CR) retards several aspects of the aging process in mammals, including age-related mortality, tumorigenesis, physiological decline [1] and the establishment of age-related transcriptional profiles [2]. The wide scope of these actions, and the profound metabolic and hormonal shifts induced by CR has led to efforts at identifying natural or synthetic compounds that mimic the effects of CR in the absence of overt metabolic and endocrine disturbances or reduced caloric intake. Because most age-related diseases are likely to be secondary to the aging process itself, the discovery of such compounds could have a profound public health impact by reducing disease incidence and possibly extending the quality and length of the human lifespan.

Resveratrol, a natural compound found in grapes and red wine has previously been shown to extend lifespan in *S. cerevisiae*, *C. elegans* and *Drosophila* through a SIRT1 dependent mechanism [3], [4]. However, recent studies have failed to reproduce these life extension results [5], [6], and other studies have demonstrated that the ability of resveratrol to activate yeast Sir2 or human SIRT1 is substrate-specific *in vitro* [7] and resveratrol has no effect on Sir2 activity *in vivo* [5]. Thus, the effects and mechanisms of resveratrol in life extension in invertebrates are currently unclear. Recently, mice fed a high fat diet supplemented with high levels of resveratrol (22 or 186 mg kg^−1^ day^−1^) were shown to have extended lifespan as compared to controls, and several metabolic alterations similar to what is observed with CR, including markers of increased mitochondrial biogenesis [8], [9]. Because control animals in this study had early mortality due to toxicity of the high fat diet, the role of resveratrol in the aging process in mammals is also unclear and a detailed examination of the physiological impact of resveratrol on aging and health parameters is indicated.

Global gene expression profiling can be used to evaluate the biological age of a tissue, because as shown for animals subjected to CR, such profiles correlate with biological as opposed to chronological age [2]. Given that compounds that retard aging may do so in a tissue-specific manner, we and others have used gene expression profiling as a method to search for compounds that can mimic the effects of CR [10], [11]. This study aimed to examine the role of resveratrol in gene expression profiles associated with mammalian aging and the specific effects of both resveratrol and CR on gene expression patterns in multiple tissues. We also compared the effects of resveratrol and CR in insulin signaling, oxidative damage, age-related cardiac function, and spontaneous tumorigenesis.

## Results

### Global impact of CR and resveratrol on age-related changes in gene expression

We fed individually-housed male (C57BL/6×C3H/He)F_1_ hybrid mice one of three diet formulations: a control diet (84 kcal mouse^−1^ week^−1^), a CR diet (63 kcal mouse^−1^ week^−1^) and a control diet supplemented with trans-resveratrol (4.9 mg kg^−1^ day^−1^) starting at middle age (14 months of age). We have previously reported that this CR regimen started at middle age in this strain leads to a 13% increase in average and maximum lifespan [10]. As expected, long-term feeding of the CR diet resulted in a significant reduction in body weight (for control mice, average body weight 35 g; for CR mice average body weight 24 g; *P*<0.01, Supplemental Figure S1); this reduction in body weight was not observed in resveratrol-fed animals (Supplemental Figure S1). Mice were sacrificed at 30–31 months of age and tissues collected for gene expression analysis. We focused our analysis on three tissues: heart, skeletal muscle and brain (neocortex).

**Figure 1:**
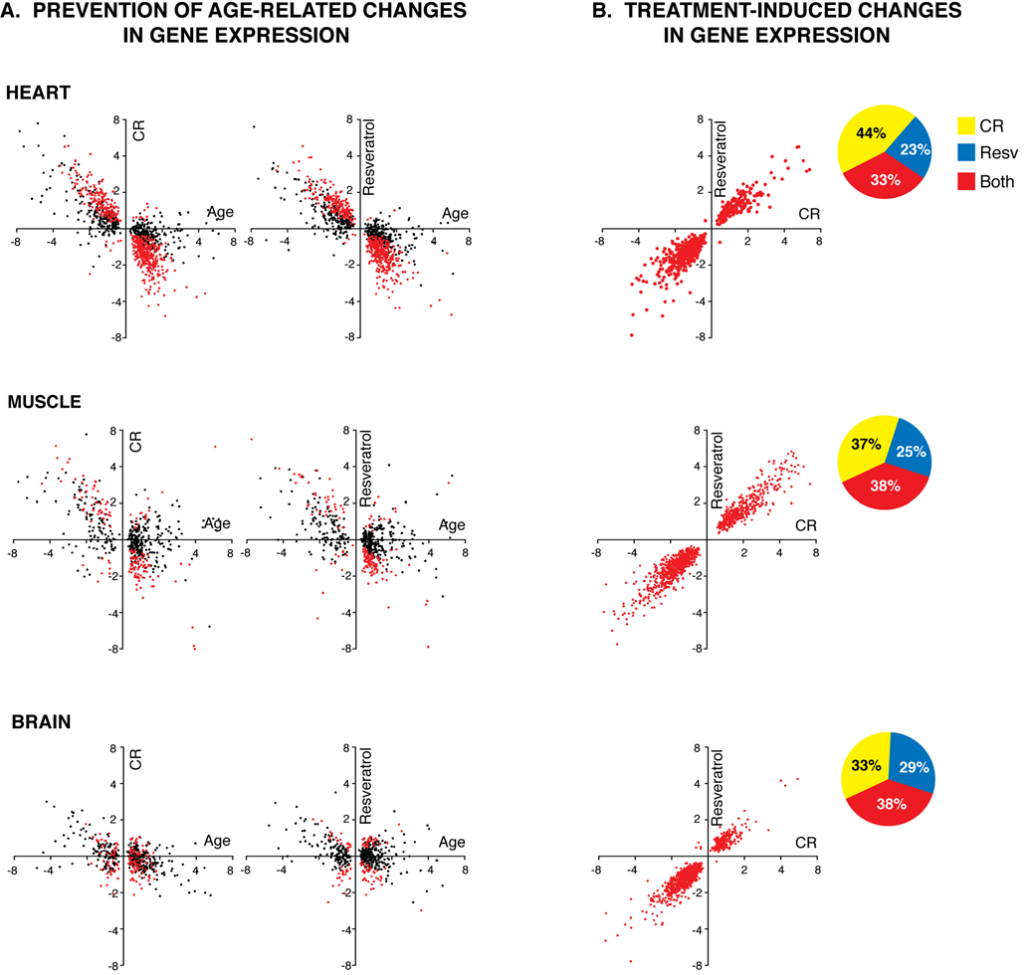
Global effects of resveratrol and CR on gene expression. Gene expression profiling of 20,687 unique transcripts using Affymetrix Mouse Genome 430 2.0 arrays was performed in young (5-months old) control and old control, CR and resveratrol fed animals (30-months old, all groups). (A). A panel of genes corresponding to significant (*P≤*0.01) changes in gene expression in the comparison between young and old control groups was examined in both CR (left) and resveratrol fed mice (right). Numbers on X and Y axes represent fold changes in the young vs. old comparison (X axis), and the old CR vs old control (Y axis, left graphs) or old resveratrol vs. old control group (Y axis, right graphs). Each dot corresponds to an specific gene. Red dots correspond to genes that are significantly altered in expression at *P≤*0.01 in both aging and CR, or aging and resveratrol comparisons. Opposite fold changes in this analysis represent prevention of aging changes. (B). A panel of genes significantly changed by CR and resveratrol (but not by age) as compared to old control mice (*P≤*0.01) is plotted. Similar fold changes in this analysis represent resveratrol mimicry of CR. Pie charts represent the proportion of genes changed by CR only, resveratrol only or both treatments. Gene expression changes plotted in graphs correspond to the genes changed significantly expression by both treatments (red).

To examine the age-associated changes in gene expression we compared transcriptional profiles of young and old mice fed the control diet. There were 1,029 genes (*P*≤0.01) that were significantly changed in expression with age in the heart. We next examined the impact of CR on the expression of these genes by comparing 30-month old CR mice and 30-month old control mice. Age-related changes in gene expression that are prevented by CR would be expected to show opposite directions of change (upregulation vs. downregulation). As previously reported [2], we observed a large effect of CR in opposing age-related changes: CR reduced 921 (90%) age-related alterations in gene expression and 536 of these represented highly significant differences (*P*≤0.01) in expression between the old control and old CR groups (Figure 1A). To examine the effect of resveratrol supplementation on age-related gene expression patterns, we performed a similar analysis comparing 30-month old control mice and 30-month old resveratrol fed animals (Figure 1A). Surprisingly, resveratrol opposed 947 (92%) of age-related changes in gene expression, and 522 of these represented highly significant differences in expression between the old control and old resveratrol groups (*P*≤0.01). Thus, resveratrol at doses that can be readily achieved through dietary supplementation in humans is as effective as CR in opposing the majority of age-related transcriptional alterations in the aging heart. Because the collection of such alterations in gene expression is a biomarker of aging, our results imply that similar to CR, middle-age onset resveratrol supplementation at low doses is likely a robust intervention in the retardation of cardiac aging. Lesser effects on aging inhibition by CR and resveratrol were obtained in skeletal muscle; aging resulted in alteration of expression of 515 skeletal muscle genes, 136 (26%) of which were significantly (*P*≤0.01) opposed by CR and resveratrol (Figure 1A). In neocortex, CR and resveratrol significantly inhibited only 19 and 13% respectively of the 505 highly significant age-related changes in gene expression (Figure 1A).

### CR and resveratrol have overlapping effects on alterations in gene expression in multiple tissues

We next examined the transcriptional profiles of CR and resveratrol fed mice for genes that are impacted by the dietary interventions, but are not altered in expression with aging. Such changes in gene expression represent shifts induced by the dietary interventions and can provide clues to mechanisms of action and the degree and nature of CR mimicry by resveratrol. We identified 747 genes that were significantly altered in expression by both CR and resveratrol in the heart (*P*≤0.01) but were not altered in expression as a result of normal aging. Strikingly, 745 (99.7%) of these gene expression alterations occurred in the same direction for both treatments (Figure 1B). This finding was also observed in muscle (1164/1164 genes, Figure 1B) and brain (1129/1134 genes, Figure 1B). Clearly, resveratrol can mimic a large component of the transcriptional profile of CR in all tissues examined.

### Resveratrol and CR prevent age-related cardiac dysfunction

**Figure 2:**
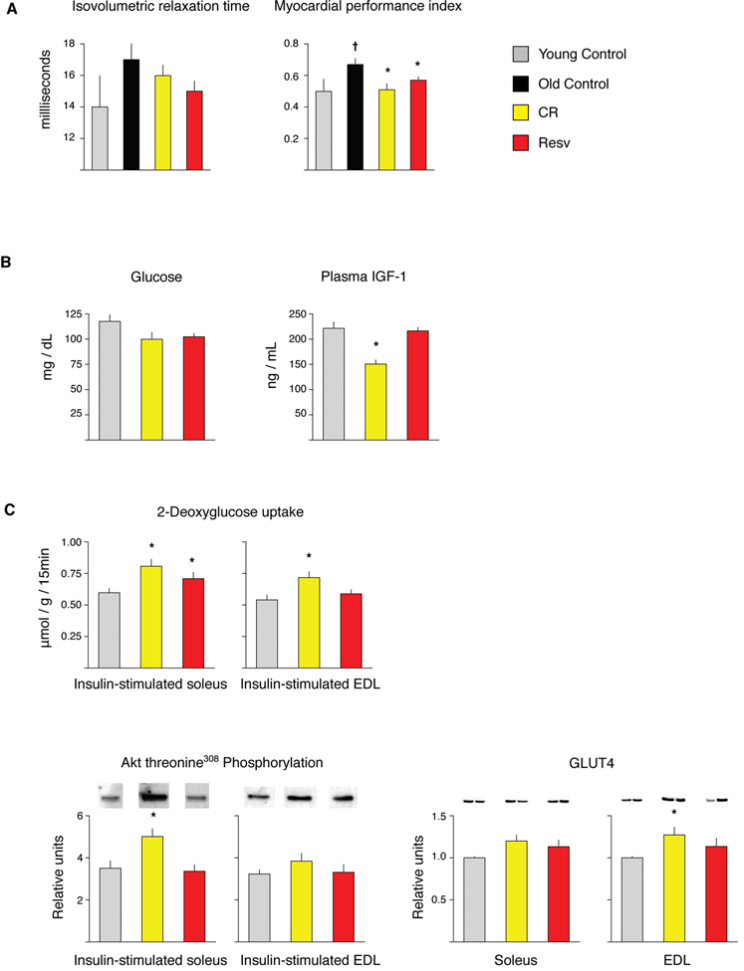
Effects of CR and resveratrol on physiological parameters. (A) Cardiac function as determined by transthoracic echocardiography (n = 7, YC, n = 10 OC, n = 8 CR, n = 9 Resv) (B) Serum glucose and plasma IGF-1 in control, CR and resveratrol treated mice (C) Glucose uptake and insulin signaling in control, CR and resveratrol fed animals (n = 16). Rate of 2-deoxyglucose uptake and Akt threonine^308^ (T^308^) phosphorylation were determined in isolated paired soleus and extensor digitorum longus (EDL) muscles with or without 0.36 nM insulin. Data were analyzed by two-way ANOVA, and the source of significant variance was tested using Student-Newman-Keuls post-hoc test (n = 15–16 muscles per group). There were no differences between groups at baseline so values shown reflect insulin-stimulated values only. GLUT4 protein abundance was determined in soleus and EDL muscles by western blotting (n = 16 muscles per group). Data were analyzed by one-way ANOVA on ranks, and the source of significant variance was tested using Dunn's post-hoc test.

Cardiac function is known to decline with age in mice and humans, a factor that likely contributes to the fact that cardiac disease is one of the leading contributors to age-related disability and death. To assess left ventricular (LV) systolic and diastolic function as well as structure, we used *in vivo* M-mode and Doppler echocardiography to examine cardiac function in young (five month-old) control mice and 25 month-old control, CR and resveratrol fed mice. Isovolumic relaxation time, a measure of diastolic function, was increased in aged animals (Figure 2A), consistent with the impaired LV relaxation that occurs with normal aging in rodents and humans [12]. Both CR and resveratrol supplementation reduced the age-related increase in this parameter, though these changes were not statistically significant. We also examined the myocardial performance index, a parameter that provides an overall assessment of cardiac function [13]. An increased index value is associated with reduced cardiac performance, and this value increased significantly with age (Figure 2A). Both CR and resveratrol supplementation almost completely prevented the age-related decrease in this parameter. Thus, resveratrol mimics the effects of CR to prevent cardiac aging at both the transcriptional and functional levels.

### Effects of resveratrol and CR on endocrine status and glucose metabolism

In an attempt to uncover the mechanisms of action of resveratrol in retarding aging parameters and its ability to mimic CR, we examined pathways implicated in aging and CR. In mammals, spontaneous mutations that result in growth hormone (GH) deficiency, such as the Prop-1 and Pit-1 dwarf mice [14], [15], or targeted mutations in the insulin-like growth factor 1 (IGF-1) receptor gene [16] or the insulin receptor substrate 1 (IRS1) can result in increased lifespan [17], suggesting an important role for IGF-1 in longevity. Some of the phenotypes that are observed in animals with altered IGF-1 or insulin signaling are also observed in CR mice, such as reduced levels of IGF-1, insulin and glucose [18]. To investigate if resveratrol inhibits transcriptional profiles of aging and mimics CR through an endocrine mechanism, we measured glucose, T3, insulin, IGF-1 and GH in five month-old control, CR and resveratrol fed mice. Following two months of dietary intervention, we observed reduced IGF-1 levels in CR mice, but not in resveratrol treated mice (Figure 2B). We did not observe significant alterations in any other hormones examined. Surprisingly, because we observed a large overlap of transcriptional shifts induced by resveratrol and CR in all organs examined, our findings also suggest that a large component of the transcriptional program induced by CR may be independent of CR-mediated alterations in plasma IGF-1, or insulin. This conclusion is supported by the finding that dwarfism and CR may impact lifespan through independent mechanisms [19], and the finding that GH deficiency and CR display minimal overlap at the gene expression level [20].

Genetic alteration of insulin related pathways has clearly been implicated in aging retardation in multiple organisms, and earlier studies have demonstrated that mice consuming a CR diet [21] or supplemented with resveratrol have improved insulin sensitivity [8], [9]. Both CR and resveratrol supplementation lowered blood glucose levels (*P* = 0.06 for CR, *P* = 0.07 for resveratrol; Figure 2B). Neither diet lowered plasma insulin (data not shown). We next examined 2-deoxyglucose (2-DG) uptake in isolated soleus and extensor digitorum longus (EDL) muscles from mice fed CR or resveratrol diets from three to five months of age. CR resulted in a significant increase in 2-DG uptake in the insulin-stimulated soleus (35%) and EDL (32%) muscles, without any effect on 2-DG uptake in the basal condition. There was also an increase in 2-DG uptake in the insulin-stimulated soleus (18%), but not in the EDL, of resveratrol treated mice. Akt phosphorylation, which partially mediates enhanced insulin sensitivity of muscle [22], was increased with CR but not with resveratrol supplementation (Figure 2C). Levels of GLUT4, the major glucose transporter in muscle, were also increased in CR, but not resveratrol-treated mice (Figure 2B). These findings suggest that the mechanism of the increased glucose uptake in insulin-stimulated skeletal muscle in resveratrol treated mice is different from that of CR mice, as neither Akt phosphorylation nor GLUT4 content were elevated by resveratrol.

### CR and resveratrol do not alter SIRT1 levels, and CR but not resveratrol induces PGC-1α transcriptional targets

**Figure 3:**
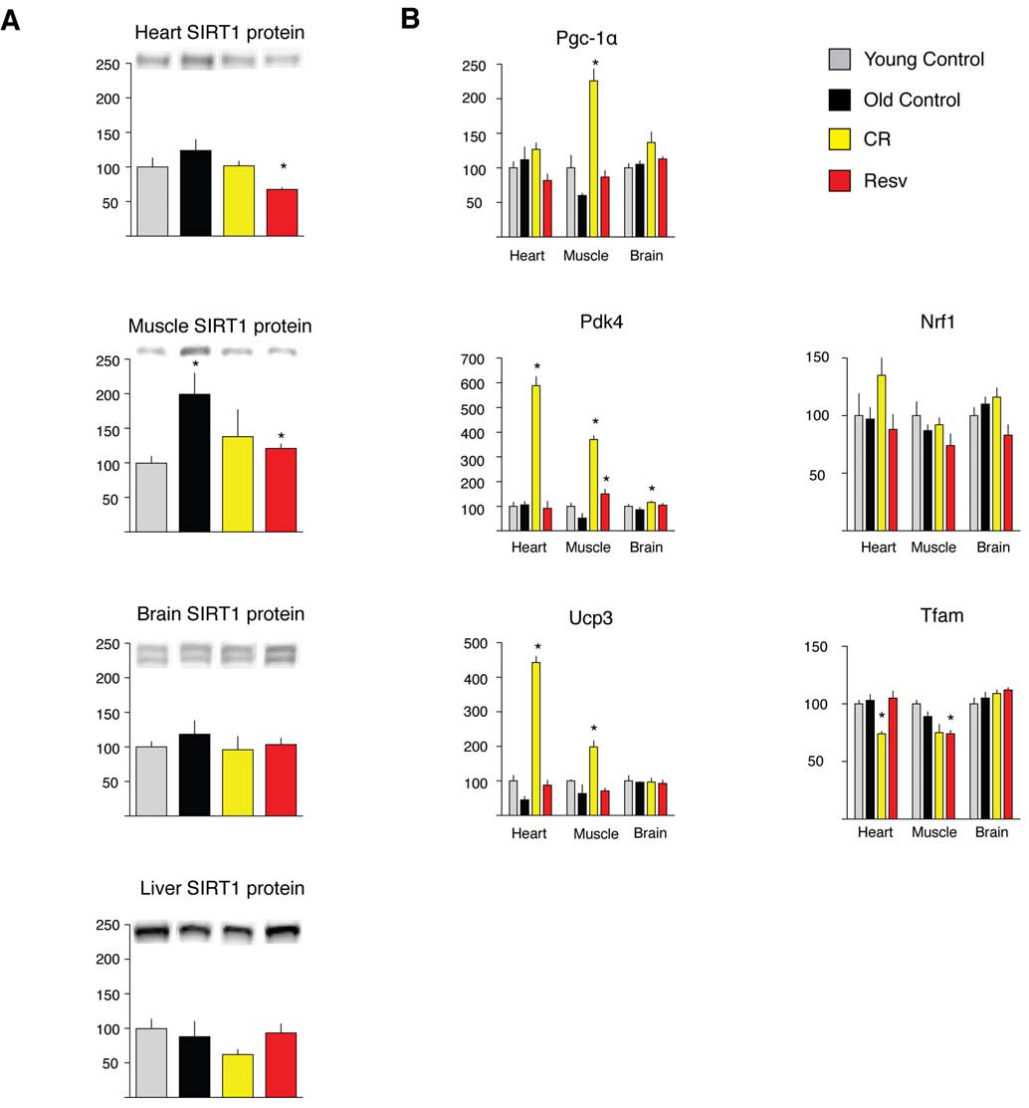
SIRT1 levels and Pgc1-α transcriptional activity in response to CR and resveratrol. (A) SIRT1 levels in liver, skeletal muscle and brain (n = at least four animals per group) were determined by Western blot analysis. A loading correction factor based on HSP70 band intensity data was used to normalize the SIRT1 band intensity data. (B) mRNA abundance for known Pgc-1α transcriptional targets is shown for heart, muscle and brain. Data on Y axis represent percentage changes relative to young controls. Results represent n = 5, values in bar graphs are means and SE. * *p*<0.01.

Feeding high levels of resveratrol to mice has been shown to be associated with increased SIRT1 activity as measured by PGC-1α acetylation and induction of its transcriptional targets [8], [9]. Because sirtuin overexpression increases longevity in some organisms and may mediate some of the effects of CR [3], [4], the induction of SIRT1 activity has been postulated to mediate the health benefits of resveratrol [8], [9]. We investigated if alterations in the levels of SIRT1 or induction of PGC-1α transcriptional activity can explain the observation that a low dose of resveratrol mimics CR. In contrast to previous finding in rats [23] and humans [24], neither CR nor resveratrol feeding significantly altered the levels of SIRT1 protein in brain or liver of mice, and SIRT1 abundance was actually significantly decreased in both heart and muscle of CR mice (Figure 3A). However, CR clearly increased the mRNA levels of Pgc-1α in skeletal muscle. CR also stimulated an increase in Pgc-1α transcriptional targets Pdk4 and Ucp3 in heart and skeletal muscle. In contrast, resveratrol did not significantly increase the levels of Pgc-1α expression or any of its transcriptional targets, with the exception of a small effect on the expression of Pdk4 in skeletal muscle (Figure 3B). Thus, our microarray results suggest that a low dose of dietary resveratrol induces a transcriptional program similar to CR in multiple tissues and retards aging parameters, but these effects may be largely independent of the increase in SIRT1 activity and activation of Pgc-1α transcriptional targets reported previously for mice in a high fat diet fed high levels of resveratrol [8], [9].

### Effects of CR and resveratrol feeding on oxidative stress parameters

Because resveratrol has been shown to act as an antioxidant both *in vitro* and *in vivo*, we tested the hypothesis that resveratrol supplementation retards aging through a reduction in oxidative damage which has been postulated to be causal in both aging and a number of age-related diseases. To test if resveratrol has an impact on spontaneous oxidative damage, we measured F2-isoprostanes, a marker of lipid peroxidation in heart, skeletal muscle and brain of mice fed CR or resveratrol diets from three to five months of age (Supplemental Figure 2). Surprisingly, we found that resveratrol significantly raised the levels of F2-isoprostanes in the heart relative to controls (*P*<0.05) and isoprostane levels in the brain were higher in resveratrol treated mice compared CR mice (*P*<0.01); isoprostane levels were similar among groups in muscle. We next examined oxidative damage to DNA and RNA in the same mice; levels of 8-hydroxy-2′-deoxyguanosine (8-OHdG) and 8-hydroxyguanosine (8-OHG) were similar among groups for each tissue (Supplemental Figure S2). Thus, our observations do not indicate lowering of spontaneous levels of oxidative damage by either CR or resveratrol.

### Functional analysis of CR and resveratrol-mediated gene expression changes in multiple tissues

**Figure 4:**
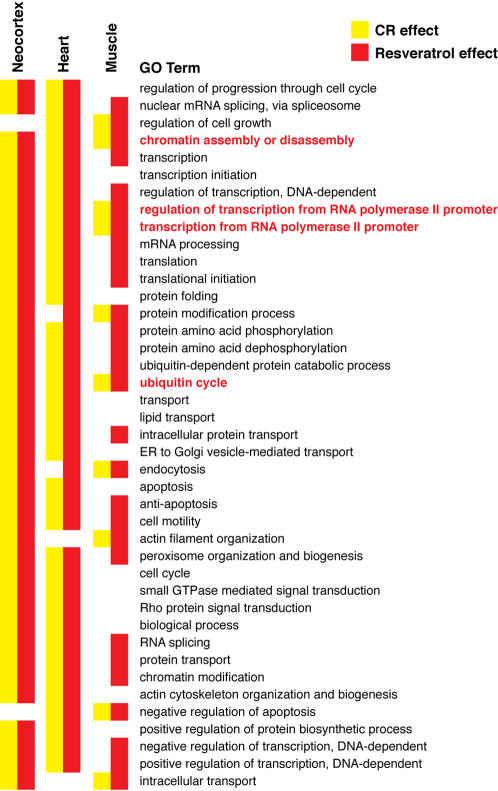
Functional gene expression analysis of CR and resveratrol fed mice using SAFE. A class matrix, which describes the functional categories and specifies what genes are members of what classes, was based on Gene Ontology (GO), and included classes with at least 10 genes, for a total of 571 classes. The SAFE analysis was then run using the default settings for the local statistic (Student's *t*) and GO terms that differed at P≤0.05 were considered significantly different. Only classes that show a significant effect for at least one treatment in one tissue are shown. Significance values for all functional classes are shown in Supplemental Table S1. Classes highlighted in blue were changed by both CR and resveratrol in all tissues examined.

**Figure 5:**
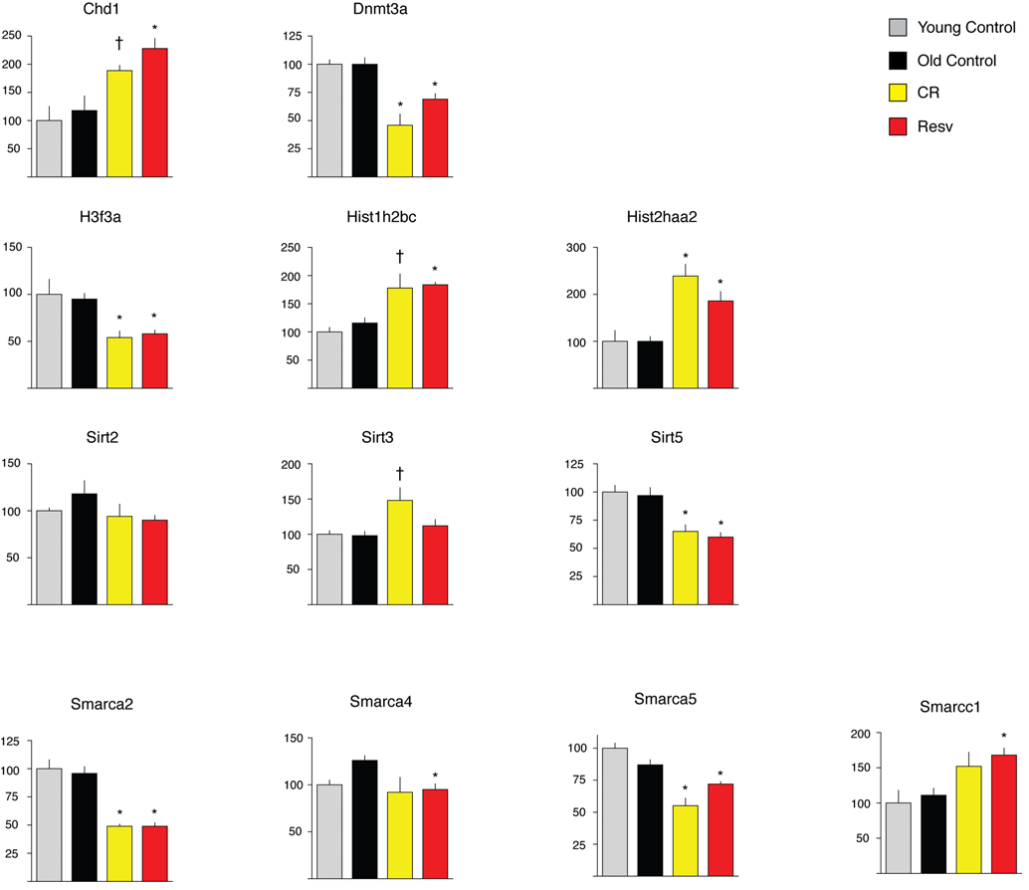
Selected genes in skeletal muscle associated with GO:0006333 (chromatin assembly and disassembly). Means identified with † were significantly different from age-matched controls at p<0.05; means identified with * were significantly different from age-matched controls at p<0.01.

Because resveratrol did not impact well-known factors that are postulated to impact aging (IGF-1, insulin, Sirt1, oxidative stress), we analyzed the gene expression data for functional categories that may be affected with age, CR or resveratrol. We used the significance analysis of function and expression (SAFE), a two-stage, permutation-based method that can be applied to various experimental designs, accounts for the unknown correlation among genes and enables permutation-based estimation of error rates [25]. We used the Gene Ontology (GO) Biological Process classification scheme for annotating gene function and only included GO terms that were represented by least 10 genes. This resulted in 571 GO terms represented in each tissue (Supplemental Table S1). Because our global analysis of age-independent shifts in gene expression indicated that resveratrol mimics CR in all tissues examined, we were particularly interested in functional classes that were significantly altered by both interventions across tissues. Only four GO terms were impacted by both CR and resveratrol across all tissues (Figure 4), and these were chromatin assembly or disassembly (GO:0006333), regulation of transcription from RNA Polymerase II promoter (GO:0006357), transcription from RNA polymerse II promoter (GO:0006366), and ubiquitin cycle (GO:0006512). Analysis of individual genes within these classes suggests that both CR and resveratrol have a major impact on expression of genes that play important roles in chromatin remodeling that defines the transcriptional/epigenetic state of the cell. In skeletal muscle (Figure 5), these include the *de novo* methylase Dnmt3a, the chromodomain helicase DNA binding protein 1 (Chd1), the sirtuin Sirt5, and the SWI/SNF complex members Smarcc1, Smarca4, Smarca5 and Smarca2. The Smarca2 gene encodes Brahma, the key component of the SWI/SNF complex, a master transcriptional switch that directs specific cellular programs and uses ATP hydrolysis to remodel chromatin [26]. Additionally, multiple histone encoding genes were altered in expression by both CR and resveratrol. Alteration in expression of the same or related genes was also observed in heart and brain.

## Discussion

The importance of chromatin remodeling proteins in defining the chromatin state of the cell, basal transcription and their ability to influence DNA repair [27] suggests a common mechanism of action for CR and resveratrol. Based on the functional analysis of gene expression profiles in multiple tissues we postulate that both CR and resveratrol impact pathways that determine chromatin remodeling, perhaps in response to a metabolic stress signal. The ensuing alteration in chromosome architecture and transcription may facilitate pathways that maintain genomic stability, or prevent epigenetic alterations, and therefore retard some aspects of the aging process in the long-term. Understanding the pathways that influence the expression of genes involved in chromatin remodeling and transcription in response to both CR and resveratrol may therefore provide key insights into the molecular basis of aging in mammals.

Our studies suggest that dietary consumption of a low dose of resveratrol partially mimics CR and inhibits some aspects of the aging process. In long lived rodent strains and in humans, lifespan is often limited by spontaneous tumorigenesis. Studies have determined that the ability of CR to inhibit spontaneous tumorigenesis is linked to the CR-mediated reduction in circulating IGF-1 [28], and in the case of mammary carcinogenesis can be reversed by the administration of IGF-1 to CR animals [29]. Our study design involved the use of a long-lived F1 hybrid mouse strain, and sacrificing mice at 30-months of age, therefore we were unable to evaluate effects of resveratrol on average or maximum lifespan. We note that unlike CR, resveratrol did not reduce circulating IGF-1 levels (Figure 2B), and there was also no decrease in spontaneous tumors at the time of sacrifice (Supplemental Table S2). In particular, spontaneous liver tumors were abundant in mice fed the control diet or resveratrol, but rare in CR mice. Thus, although a low dose of resveratrol can improve quality of life by retarding aging parameters such as cardiac dysfunction, a nutritional or pharmaceutical strategy to also increase lifespan in mice will likely require blockage of the IGF-1 axis or its targets.

Our study also raises questions regarding proposed mechanisms of action of both CR and resveratrol. Findings from previous studies performed with higher doses of resveratrol in mice suggested that an increase in SIRT1 activity and the resulting deacetylation of the transcriptional coactivator Pgc-1α is a central mechanism of action. Health benefits observed included reduced mortality associated with the high-fat diet, improved motor performance and improved insulin sensitivity [8], [9]. Surprisingly, the induction of Pgc-1α transcriptional targets was observed in skeletal muscle but not heart [9]. Given that resveratrol is known to be cardioprotective in models of ischemia-reperfusion [30], and our own findings of strong activity in the retardation of cardiac aging, but no effects on Pgc-1α transcriptional targets, it seems unlikely that SIRT1/Pgc-1α play a role in resveratrol's cardiac effects. It is likely that the effects of resveratrol feeding at lower doses reported here are distinct than those observed with higher doses, with particular relevance to the induction of SIRT1 activity. We also did not find support for the hypothesis that induction of SIRT1 directly mediates the effects of CR in the tissues examined, since SIRT1 levels were not altered at the mRNA or protein levels. Previous studies in rats [23] and humans [24] suggest that CR induces SIRT1 in these species, but the only study in mice that addressed this issue used an “every other day” feeding protocol, and therefore does not represent CR [31]. To our knowledge our study is the first to attempt to detect an induction of SIRT1 in CR mice, and we have not observed such induction in any of the tissues examined (heart, liver, skeletal muscle and brain). Given that overexpression of SIRT1 in mice does induce physiological alterations consistent with CR [32], it is possible that CR impacts the levels of SIRT1 and other sirtuins in other tissues that play a central role in regulating metabolism, such the pancreas [33], [34]. We also note that despite the absence of SIRT1 induction, our findings are consistent with a general alteration in the expression of genes involved in chromatin remodeling by CR and resveratrol, including other sirtuins (Figure 5). Other proposed biochemical mechanisms of action of resveratrol that were not examined in this study may include stimulation of AMP kinase [35], and increased nitric oxide synthase activity [36]–[38]. We also note that studies suggest that the nutrient sensor target of rapamycin (TOR) pathway, involved in the regulation of growth and autophagy mediates the life-extension effects of CR in *Drosophila* [39] and *C. elegans* [40], and therefore may play a role in the resveratrol effects reported here. Because resveratrol mimics CR at the gene expression level, but did not mimic the effects of CR on the few proteins examined in this study (GLUT4, AKT, IGF-1 and SIRT1) it is possible that although similar at the transcriptional level, CR and resveratrol have different effects with regard to translational regulation. An examination of several proteins encoded by genes affected in expression by both CR and resveratrol should clarify this issue.

Our findings that a low dose of resveratrol partially mimics CR at the gene expression level and leads to prevention of some age-related parameters suggests that clinical trials with resveratrol should be conducted to test the relevance of these findings to humans. Because cardiac disease is a major contributor to age-related mortality, positive findings could lead to a novel and important approach to improve the quality of human life.

## Materials and Methods

### Animals and diets

Male C57BL/6×C3H/He F1 hybrid mice were purchased at six weeks of age (Harlan-Teklad), individually housed in a specific pathogen free facility and fed a control diet based on the AIN-93M formulation (Bio-Serv, Frenchtown, NJ). Upon arrival and until 14 months of age, mice received 84 kcal per week, approximately 10% lower than ad lib intake. Starting at 14 months of age, mice were randomly divided into one of three groups: a Control group receiving the same diet; a resveratrol-treated group receiving the same diet as the controls but supplemented with 50 mg resveratrol per kg diet (resveratrol purchased from Sigma Chemical, St. Louis); this dose is approximately equal to 4.9 mg resveratrol per kg body weight assuming a 35 g mouse; a calorie restricted (CR) group receiving 63 kcal per week. Details on feeding Control and CR diets have been published elsewhere [41]. At 30 months of age, tissues were collected from mice, flash-frozen in liquid nitrogen and stored at −80°C. Tissues were also collected from a group of young control mice that received the control diet from two to five months of age. The number of spontaneous deaths in each cohort prior to the age of sacrifice was 2/30, 0/18 and 2/22 in the control, CR and resveratrol-treated groups, respectively (Supplemental Table S2). Studies in young mice were performed as described above except the CR or resveratrol treatments were begun at two months of age and were continued through five months of age. All procedures complied with the Institutional Animal Care and Use Committee of the William S. Middleton Memorial Veterans Hospital, Madison, WI

### Gene expression profiling/Functional Classification

Gene expression profiling was performed using Affymetrix Mouse Genome 430 2.0 arrays as described previously [2]. Aging comparisons were made between Young and Old Control groups; CR and resveratrol effects were determined by comparing treated mice to the Old Control group. Genes were considered to be significantly different at *P*≤0.01 using Student's *t*-tests.

To discover gene classes that are significantly different between the two groups in each comparison, we used the Significance Analysis of Function and Expression (SAFE) analysis [25]. This approach is a two-stage, permutation-based method that can be applied to various experimental designs, accounts for the unknown correlation among genes and enables permutation-based estimation of error rates. The SAFE analysis requires three input data structures: the gene expression data, a response vector, and a class matrix. The first two were created from the array data using custom source code to reformat the data to conform to what SAFE expects. The class matrix, which describes the functional categories and specifies what genes are members of what classes, was created in a similar way and was based on Gene Ontology (GO), and included classes with at least 10 genes. This resulted in 571 classes. The SAFE analysis was then run using the default settings for the local statistic (Student's *t*) and GO terms that differed at P≤0.05 were considered significantly different.

### Cardiac function

Transthoracic echocardiography was performed using an Acuson Sequoia (Siemens) ultrasonograph with a 15-MHz transducer. For acquisition of two-dimensional guided M-mode images at the tips of papillary muscles and Doppler studies, mice were sedated by IP administration of 100 mg/kg ketamine and maintained on a heated platform in a left lateral decubitus position. The chest was shaved and prewarmed coupling gel applied. Transmitral velocities were measured using Doppler pulse wave imaging. All images were saved to an on-board optical disk. Echocardiograms were performed and analyzed by an investigator blinded to the experimental groups.

End diastolic and systolic left ventricular (LV) diameter as well as anterior and posterior wall (AW and PW respectively) thicknesses were measured on line from M-mode images using the leading edge-to-leading edge convention. All parameters were measured over at least three consecutive cardiac cycles and averaged. Left ventricular fractional shortening was calculated as [(LV diameter_diastole_ –LV diameter_systole_ )/LV diameter_diastole_] × 100 and LV mass was calculated by using the formula. Relative wall thickness was calculated as 2× Posterior wall_diastole_/LV diameter_diastole_. Heart rate was determined from at least three consecutive intervals from the pulse wave Doppler tracings of the LV outflow tract. Isovolumic relaxation time was measured as the time from the closing of the aortic value to the opening of the mitral value from pulse wave Doppler tracings of the LV outflow tract and mitral inflow region. Myocardial Performance Index (MPI) was calculated as (a-b)/b where a = mitral valve closure time and b = aortic injection time

### Glucose uptake and insulin signaling

Rate of 2-deoxyglucose uptake and Akt threonine^308^ (T^308^) phosphorylation were determined in isolated paired soleus and extensor digitorum longus (EDL) muscles at baseline with or without 0.36 nM insulin. Data were analyzed by two-Way ANOVA, and the source of significant variance was tested using Student-Newman-Keuls post-hoc test (n = 15–16 muscles per group). There were no differences between groups at without insulin so values shown reflect insulin-stimulated values only.

GLUT4 protein abundance was determined in soleus and EDL muscles by western blotting (n = 16 muscles per group). Data were analyzed by one-way ANOVA on ranks, and the source of significant variance was tested using Dunn's post-hoc test.

### SIRT1 abundance

Samples for western blotting were electrophoresed in polyacrylamide tris-acetate gels (Invitrogen) and transferred to nitrocellulose. Membranes were blocked in 0.5% gelatin in TBS-T for one hour. Primary and secondary antibodies were diluted in 0.5% gelatin in TBS-T. Rabbit anti-SIRT1 was purchased from Upstate (Charlottesville, VA), goat anti-HSP70 and horseradish peroxidase (HRP)-linked donkey anti-goat IgG were purchased from Santa Cruz Biotechnology (Santa Cruz, CA). HRP-linked goat anti-rabbit IgG was purchased from Pierce (Rockford, IL). Chemiluminescent bands were visualized and analyzed using a UVP Bioimaging Systems (Upland, CA) EpiChemi II and LabWorks Image Acquisition and Analysis Software. HSP70 was used as a loading control. A loading correction factor based on the HSP70 band intensity data was used to adjust the SIRT1 band intensity data.

### Isoprostanes/nucleic acid damage

Isoprostanes were quantified in skeletal muscle tissue utilizing a highly precise and accurate method employing stable isotope dilution mass spectrometry as described previously [42].

RNA and DNA were simultaneously isolated from tissues by homogenization in 3M guanidine isothiocyanate (GTC) followed by a phenol-chloroform extraction procedure with inclusion of the metal chelator desferoxamine. Samples were hydrolyzed using nuclease P_1_ and alkaline phosphatase into single nucleosides and analyzed using a highly sensitive HPLC/EC/UV system (Coulochem III, ESA Inc., Chelmsford, MA). Samples were compared against a calibration curve with known standards to quantify the levels of the oxidative products 8-hydroxy-2′-guanosine/10^6^ 2′-guanosine (RNA) or 8-hydroxy-2′-deoxyguanosine/10^6^ 2′-deoxyguanosine (DNA).

## Supporting Information

Table S1 Complete list of Gene Ontology Biological Processes from the SAFE analysis
(0.11 MB PDF)

Table S2 Necropsy results from old mice fed a control, CR or resveratrol-supplemented diet
(0.04 MB PDF)
