# Correction: A Low Dose of Dietary Resveratrol Partially Mimics Caloric Restriction and Retards Aging Parameters in Mice

**DOI:** 10.1371/annotation/8333176c-b08c-4dfb-a829-6331c0fc6064

**Published:** 2008-06-13

**Authors:** Jamie L. Barger, Tsuyoshi Kayo, James M. Vann, Edward B. Arias, Jelai Wang, Timothy A. Hacker, Ying Wang, Daniel Raederstorff, Jason D. Morrow, Christiaan Leeuwenburgh, David B. Allison, Kurt W. Saupe, Gregory D. Cartee, Richard Weindruch, Tomas A. Prolla

The gene expression data reported in this paper are publicly accessible in the NCBI-GEO database at http://www.ncbi.nlm.nih.gov/geo/query/acc.cgi?acc=GSE11291 . The full list of accession numbers is presented below.

Samples (60)

GSM284967 heart-5 months of age-control diet-biological replicate1

GSM284968 heart-5 months of age-control diet-biological replicate2

GSM284969 heart-5 months of age-control diet-biological replicate3

GSM284970 heart-5 months of age-control diet-biological replicate4

GSM284971 heart-5 months of age-control diet-biological replicate5

GSM284972 heart-30 months of age-control diet-biological replicate1

GSM284973 heart-30 months of age-control diet-biological replicate2

GSM284974 heart-30 months of age-control diet-biological replicate3

GSM284975 heart-30 months of age-control diet-biological replicate4

GSM284976 heart-30 months of age-control diet-biological replicate5

GSM284977 heart-30 months of age-CR diet-biological replicate1

GSM284978 heart-30 months of age-CR diet-biological replicate2

GSM284979 heart-30 months of age-CR diet-biological replicate3

GSM284980 heart-30 months of age-CR diet-biological replicate4

GSM284981 heart-30 months of age-CR diet-biological replicate5

GSM284982 heart-30 months of age-resveratrol diet-biological replicate1

GSM284983 heart-30 months of age-resveratrol diet-biological replicate2

GSM284984 heart-30 months of age-resveratrol diet-biological replicate3

GSM284985 heart-30 months of age-resveratrol diet-biological replicate4

GSM284986 heart-30 months of age, resveratrol diet-biological replicate5

GSM284987 gastrocnemius-5 months of age-control diet-biological replicate1

GSM284988 gastrocnemius-5 months of age-control diet-biological replicate2

GSM284989 gastrocnemius-5 months of age-control diet-biological replicate3

GSM284990 gastrocnemius-5 months of age-control diet-biological replicate4

GSM284991 gastrocnemius-5 months of age-control diet-biological replicate5

GSM284992 gastrocnemius-30 months of age-control diet-biological replicate1

GSM284993 gastrocnemius-30 months of age-control diet-biological replicate2

GSM284994 gastrocnemius-30 months of age-control diet-biological replicate3

GSM284995 gastrocnemius-30 months of age-control diet-biological replicate4

GSM284996 gastrocnemius-30 months of age-control diet-biological replicate5

GSM284997 gastrocnemius-30 months of age-CR diet-biological replicate1

GSM284998 gastrocnemius-30 months of age-CR diet-biological replicate2

GSM284999 gastrocnemius-30 months of age-CR diet-biological replicate3

GSM285000 gastrocnemius-30 months of age-CR diet-biological replicate4

GSM285001 gastrocnemius-30 months of age-CR diet-biological replicate5

GSM285002 gastrocnemius-30 months of age-resveratrol diet-biological replicate1

GSM285003 gastrocnemius-30 months of age-resveratrol diet-biological replicate2

GSM285004 gastrocnemius-30 months of age-resveratrol diet-biological replicate3

GSM285005 gastrocnemius-30 months of age-resveratrol diet-biological replicate4

GSM285006 gastrocnemius-30 months of age, resveratrol diet-biological replicate5

GSM285007 neocortex-5 months of age-control diet-biological replicate1

GSM285008 neocortex-5 months of age-control diet-biological replicate2

GSM285009 neocortex-5 months of age-control diet-biological replicate3

GSM285010 neocortex-5 months of age-control diet-biological replicate4

GSM285011 neocortex-5 months of age-control diet-biological replicate5

GSM285012 neocortex-30 months of age-control diet-biological replicate1

GSM285013 neocortex-30 months of age-control diet-biological replicate2

GSM285014 neocortex-30 months of age-control diet-biological replicate3

GSM285015 neocortex-30 months of age-control diet-biological replicate4

GSM285016 neocortex-30 months of age-control diet-biological replicate5

GSM285017 neocortex-30 months of age-CR diet-biological replicate1

GSM285018 neocortex-30 months of age-CR diet-biological replicate2

GSM285019 neocortex-30 months of age-CR diet-biological replicate3

GSM285020 neocortex-30 months of age-CR diet-biological replicate4

GSM285021 neocortex-30 months of age-CR diet-biological replicate5

GSM285022 neocortex-30 months of age-resveratrol diet-biological replicate1

GSM285023 neocortex-30 months of age-resveratrol diet-biological replicate2

GSM285024 neocortex-30 months of age-resveratrol diet-biological replicate3

GSM285025 neocortex-30 months of age-resveratrol diet-biological replicate4

GSM285026 neocortex-30 months of age, resveratrol diet-biological replicate5

