# Correction: A Low Dose of Dietary Resveratrol Partially Mimics Caloric Restriction and Retards Aging Parameters in Mice

**DOI:** 10.1371/annotation/7d56e94e-3582-413d-b987-fccd0da79081

**Published:** 2008-06-23

**Authors:** Jamie L. Barger, Tsuyoshi Kayo, James M. Vann, Edward B. Arias, Jelai Wang, Timothy A. Hacker, Ying Wang, Daniel Raederstorff, Jason D. Morrow, Christiaan Leeuwenburgh, David B. Allison, Kurt W. Saupe, Gregory D. Cartee, Richard Weindruch, Tomas A. Prolla

Supplementary Figure S1 does not appear. Please view it here:
