# Correction: A Low Dose of Dietary Resveratrol Partially Mimics Caloric Restriction and Retards Aging Parameters in Mice

**DOI:** 10.1371/annotation/c54ef754-1962-4125-bf19-76d3ec6f19e5

**Published:** 2008-06-23

**Authors:** Jamie L. Barger, Tsuyoshi Kayo, James M. Vann, Edward B. Arias, Jelai Wang, Timothy A. Hacker, Ying Wang, Daniel Raederstorff, Jason D. Morrow, Christiaan Leeuwenburgh, David B. Allison, Kurt W. Saupe, Gregory D. Cartee, Richard Weindruch, Tomas A. Prolla

The hyperlink to Supplementary Figure S2 connects to the wrong document. Please view the correct Figure S2 here:
