# Supplementary material for: A Low Dose of Dietary Resveratrol Partially Mimics Caloric Restriction and Retards Aging Parameters in Mice
Source: PLoS One. 2008 Jun 4;3(6):e2264. doi: 10.1371/journal.pone.0002264 (PMC2386967; doi:10.1371/journal.pone.0002264)
Supplement: Table S1 — Complete list of Gene Ontology Biological Processes from the SAFE analysis (0.11 MB PDF) [file pone.0002264.s001.pdf]

Supplemental Table 1. Complete list of Gene Ontology Biological Processes from the SAFE analysis

	Neocortex p-values			Heart p-values			Muscle p-values			
GO_term	Age	CR	Resv	Age	CR	Resv	Age	CR	Resv	GO_ID_SAFE
autophagic vacuole formation	0.265	0.028	0.006	0.783	0.443	0.013	0.221	0.296	0.227	45
protein import into nucleus, docking	0.751	0.024	0.095	0.471	0.115	0.516	0.505	0.026	0.110	59
protein import into nucleus, translocation	0.774	0.177	0.261	0.336	0.444	0.114	0.617	0.025	0.046	60
regulation of progression through cell cycle	0.969	0.015	0.002	0.073	0.040	0.004	0.259	0.082	0.328	74
DNA damage checkpoint	0.847	0.861	0.710	0.264	0.279	0.287	0.252	0.882	1.000	77
regulation of cyclin-dependent protein kinase activity	0.486	0.146	0.365	0.123	0.659	0.019	0.054	0.799	0.420	79
G1/S transition of mitotic cell cycle	0.679	0.207	0.387	0.650	0.010	0.852	0.993	0.142	0.538	82
G2/M transition of mitotic cell cycle	0.944	0.229	0.141	0.378	0.829	0.183	0.047	0.890	0.648	86
negative regulation of transcription from RNA polymerase II pror	0.377	0.051	0.006	0.644	0.030	0.034	0.473	0.560	0.540	122
two-component signal transduction system (phosphorelay)	0.020	0.081	0.199	0.449	0.370	0.542	0.164	0.366	0.645	160
MAPKKK cascade	0.472	0.066	0.591	0.081	0.055	0.396	0.006	0.060	0.119	165
mRNA catabolic process, nonsense-mediated decay	0.232	0.087	0.006	0.135	0.078	0.033	0.238	0.292	0.003	184
activation of MAPK activity	0.468	0.704	0.958	0.440	0.482	0.380	0.075	0.441	0.397	187
inactivation of MAPK activity	0.016	0.064	0.019	0.078	0.107	0.290	0.620	0.612	0.792	188
microtubule cytoskeleton organization and biogenesis	0.469	0.688	0.630	0.056	0.531	0.005	0.037	0.848	0.297	226
spliceosome assembly	0.924	0.018	0.176	0.045	0.661	0.519	0.524	0.508	0.049	245
peptidoglycan metabolic process	0.538	0.876	0.583	0.852	0.835	0.904	0.395	0.706	0.934	270
nuclear mRNA splicing, via spliceosome	0.922	0.010	0.006	0.023	0.015	0.032	0.159	0.078	0.003	398
telomere maintenance	0.868	0.725	0.121	0.053	0.707	0.808	0.827	0.411	0.520	723
cell morphogenesis	0.520	0.757	0.806	0.066	0.120	0.141	0.819	0.677	0.400	902
cytokinesis	0.043	0.888	0.115	0.539	0.169	0.585	0.759	0.367	0.169	910
skeletal development	0.639	0.663	0.748	0.576	0.465	0.744	0.108	0.540	0.740	1501
cartilage condensation	0.062	0.128	0.096	0.617	0.234	0.193	0.036	0.164	0.374	1502
ossification	0.730	0.520	0.733	0.092	0.617	0.214	0.046	0.006	0.241	1503
angiogenesis	0.016	0.048	0.146	0.043	0.007	0.030	0.034	0.337	0.042	1525
ovarian follicle development	0.454	0.241	0.416	0.860	0.708	0.781	0.729	0.926	0.692	1541
regulation of cell growth	0.665	0.237	0.150	0.008	0.015	0.012	0.152	0.038	0.003	1558
blood vessel development	0.265	0.369	0.387	0.169	0.045	0.293	0.424	0.060	0.012	1568
patterning of blood vessels	0.279	0.158	0.811	0.034	0.442	0.125	0.721	0.378	0.131	1569
vasculogenesis	0.180	0.198	0.605	0.038	0.092	0.706	0.516	0.309	0.305	1570
osteoblast differentiation	0.240	0.567	0.724	0.768	0.453	0.159	0.210	0.006	0.012	1649
metanephros development	0.704	0.393	0.715	0.534	0.770	0.693	0.279	0.378	0.629	1656
ureteric bud development	0.617	0.165	0.659	0.584	0.496	0.733	0.141	0.088	0.309	1657
ureteric bud branching	0.200	0.809	0.341	0.527	0.640	0.907	0.696	0.378	0.675	1658
response to hypoxia	0.361	0.054	0.055	0.007	0.067	0.005	0.658	0.701	0.734	1666
in utero embryonic development	0.960	0.278	0.030	0.007	0.047	0.005	0.232	0.299	0.570	1701
mesoderm formation	0.163	0.111	0.028	0.285	0.070	0.005	0.790	0.006	0.014	1707
cell fate specification	0.080	0.127	0.858	0.433	0.341	0.494	0.526	0.550	0.952	1708
cell fate determination	0.397	0.190	0.432	0.135	0.025	0.064	0.492	0.909	0.804	1709
eye development (sensu Mammalia)	0.262	0.122	0.233	0.512	0.520	0.213	0.164	0.042	0.705	1747
neural crest cell migration	0.297	0.050	0.196	0.587	0.571	0.725	0.550	0.020	0.103	1755
somitogenesis	0.922	0.557	0.943	0.080	0.359	0.167	0.876	0.879	0.919	1756
induction of an organ	0.868	0.428	0.928	0.888	0.831	0.950	0.492	0.560	1.000	1759
neuron migration	0.115	0.289	0.320	0.865	0.953	0.731	0.151	0.017	0.307	1764
cytokine production	0.600	0.942	0.503	0.324	0.276	0.037	0.425	0.074	0.436	1816
kidney development	0.633	0.609	0.977	0.723	0.952	0.916	0.928	0.486	0.979	1822
neural tube closure	0.094	0.302	0.090	0.189	0.187	0.592	0.503	0.371	0.462	1843
placenta development	0.984	0.610	0.718	0.837	0.621	0.775	0.982	0.749	0.509	1890
heart looping	0.990	0.368	0.223	0.029	0.122	0.305	0.577	0.617	0.591	1947
blood vessel remodeling	0.260	0.404	0.193	0.674	0.341	0.211	0.984	0.940	0.831	1974
morphogenesis of an epithelium	0.776	0.499	0.718	0.846	0.683	0.896	0.289	0.388	0.549	2009
antigen processing and presentation of peptide antigen via MHC	0.127	0.937	0.332	0.992	0.874	0.948	0.178	0.815	0.628	2474
carbohydrate metabolic process	0.760	0.321	0.035	0.008	0.035	0.030	0.046	0.752	0.690	5975
glycogen metabolic process	0.943	0.315	0.033	0.008	0.191	0.062	0.110	0.120	0.412	5977
glucose metabolic process	0.323	0.309	0.925	0.016	0.032	0.021	0.008	0.601	0.766	6006
N-acetylglucosamine metabolic process	0.579	0.418	0.473	0.070	0.582	0.135	0.559	0.903	0.817	6044
glycerol metabolic process	0.797	0.100	0.814	0.027	0.007	0.041	0.208	0.637	0.262	6071
generation of precursor metabolites and energy	0.923	0.287	0.352	0.706	0.376	0.354	0.097	0.591	0.404	6091
gluconeogenesis	0.680	0.563	0.861	0.505	0.070	0.065	0.175	0.027	0.324	6094
glycolysis	0.858	0.033	0.379	0.642	0.068	0.054	0.413	0.375	0.035	6096
tricarboxylic acid cycle	0.749	0.043	0.126	0.310	0.038	0.028	0.042	0.846	0.003	6099
electron transport	0.450	0.655	0.266	0.299	0.103	0.154	0.077	0.381	0.053	6118
mitochondrial electron transport, NADH to ubiquinone	0.192	0.043	0.325	0.266	0.301	0.246	0.430	0.523	0.014	6120
nucleobase, nucleoside, nucleotide and nucleic acid metabolic p	0.854	0.150	0.616	0.018	0.593	0.310	0.267	0.429	0.333	6139
purine nucleotide biosynthetic process	0.766	0.207	0.052	0.047	0.030	0.033	0.137	0.072	0.015	6164
cAMP biosynthetic process	0.251	0.095	0.742	0.067	0.595	0.418	0.337	0.704	0.773	6171
cGMP biosynthetic process	0.561	0.853	0.341	0.276	0.233	0.670	0.583	0.253	0.115	6182
GTP biosynthetic process	0.732	0.348	0.184	0.289	0.933	0.519	0.903	0.659	0.360	6183
UTP biosynthetic process	0.629	0.528	0.240	0.260	0.927	0.432	0.976	0.793	0.572	6228
CTP biosynthetic process	0.798	0.637	0.261	0.350	0.893	0.470	0.912	0.700	0.380	6241
DNA metabolic process	0.253	0.680	0.183	0.444	0.697	0.542	0.074	0.512	0.779	6259
DNA replication	0.261	0.160	0.013	0.057	0.007	0.013	0.625	0.632	0.340	6260
DNA-dependent DNA replication	0.379	0.420	0.833	0.451	0.122	0.537	0.266	0.312	0.146	6261
DNA topological change	0.708	0.583	0.563	0.028	0.119	0.347	0.778	0.233	0.135	6265
DNA unwinding during replication	0.757	0.810	0.518	0.364	0.474	0.215	0.828	0.205	0.413	6268
DNA replication initiation	0.104	0.566	0.273	0.247	0.424	0.322	1.000	0.395	0.439	6270
DNA repair	0.585	0.222	0.068	0.008	0.010	0.005	0.002	0.796	0.646	6281
base-excision repair	0.586	0.176	0.021	0.052	0.341	0.347	0.512	0.769	0.197	6284
nucleotide-excision repair	0.754	0.262	0.236	0.058	0.180	0.603	0.462	0.970	0.265	6289
mismatch repair	0.772	0.855	0.271	0.324	0.475	0.713	0.666	0.895	0.954	6298
double-strand break repair	0.248	0.360	0.214	0.259	0.718	0.129	0.402	0.775	0.660	6302
DNA methylation	0.692	0.306	0.773	0.059	0.581	0.177	0.601	0.438	0.328	6306
DNA fragmentation during apoptosis	0.289	0.435	0.779	0.062	0.118	0.384	0.626	0.669	0.794	6309
DNA recombination	0.757	0.477	0.094	0.075	0.294	0.129	0.049	0.088	0.753	6310
DNA packaging	0.833	0.017	0.103	0.257	0.015	0.052	0.843	0.134	0.065	6323
establishment and/or maintenance of chromatin architecture	0.310	0.099	0.056	0.703	0.086	0.753	0.921	0.308	0.065	6325
chromatin assembly or disassembly	0.310	0.022	0.006	0.033	0.036	0.027	0.161	0.016	0.045	6333
nucleosome assembly	0.977	0.184	0.282	0.402	0.187	0.172	0.572	0.392	0.091	6334
chromatin remodeling	0.433	0.144	0.045	0.178	0.035	0.069	0.924	0.133	0.209	6338
chromatin silencing	0.807	0.230	0.208	0.544	0.455	0.655	0.595	0.125	0.399	6342
transcription	0.253	0.005	0.002	0.043	0.012	0.003	0.482	0.134	0.020	6350
transcription initiation	0.305	0.028	0.023	0.138	0.007	0.036	0.807	0.220	0.541	6352
regulation of transcription, DNA-dependent	0.291	0.013	0.003	0.167	0.046	0.004	0.482	0.102	0.022	6355
regulation of transcription from RNA polymerase II promoter	0.491	0.004	0.046	0.008	0.007	0.012	0.532	0.013	0.012	6357
rRNA processing	0.504	0.016	0.173	0.161	0.067	0.021	0.653	0.460	0.211	6364
transcription from RNA polymerase II promoter	0.589	0.018	0.021	0.008	0.007	0.050	0.315	0.006	0.047	6366
transcription initiation from RNA polymerase II promoter	0.510	0.015	0.095	0.028	0.059	0.043	0.504	0.766	0.494	6367
mRNA splice site selection	0.896	0.166	0.006	0.846	0.042	0.570	0.283	0.628	0.669	6376

Supplemental Table 1. Complete list of Gene Ontology Biological Processes from the SAFE analysis

	Neocortex p-values			Heart p-values			Muscle p-values			
GO_term	Age	CR	Resv	Age	CR	Resv	Age	CR	Resv	GO_ID_SAFE
mRNA polyadenylation	0.955	0.746	0.518	0.330	0.514	0.339	0.961	0.530	0.442	6378
RNA processing	0.653	0.010	0.016	0.095	0.141	0.070	0.621	0.078	0.120	6396
mRNA processing	0.834	0.004	0.006	0.008	0.036	0.005	0.277	0.127	0.025	6397
RNA catabolic process	0.871	0.079	0.524	0.025	0.111	0.015	0.891	0.224	0.121	6401
mRNA catabolic process	0.424	0.231	0.541	0.024	0.057	0.017	0.397	0.297	0.138	6402
mRNA export from nucleus	0.828	0.235	0.018	0.046	0.137	0.206	0.094	0.905	0.070	6406
translation	0.854	0.008	0.047	0.008	0.010	0.005	0.253	0.498	0.003	6412
translational initiation	0.553	0.010	0.005	0.008	0.007	0.005	0.277	0.641	0.009	6413
translational elongation	0.862	0.067	0.006	0.023	0.089	0.033	0.037	0.630	0.014	6414
regulation of translation	0.143	0.134	0.034	0.172	0.317	0.023	0.410	0.172	0.039	6417
tRNA aminoacylation for protein translation	0.740	0.038	0.014	0.064	0.045	0.246	0.250	0.077	0.022	6418
regulation of protein biosynthesis_DUPLICATE	0.556	0.113	0.046	0.034	0.516	0.015	0.110	0.545	0.025	6445
regulation of translational initiation	0.500	0.079	0.706	0.025	0.069	0.085	0.142	0.091	0.009	6446
protein folding	0.306	0.007	0.026	0.036	0.007	0.013	0.266	0.232	0.344	6457
protein complex assembly	0.319	0.010	0.003	0.046	0.467	0.164	0.201	0.246	0.061	6461
protein modification process	0.068	0.007	0.005	0.008	0.061	0.005	0.407	0.019	0.016	6464
protein amino acid phosphorylation	0.203	0.031	0.006	0.016	0.007	0.024	0.184	0.084	0.041	6468
negative regulation of protein kinase activity	0.017	0.292	0.034	0.008	0.665	0.056	0.234	0.934	0.941	6469
protein amino acid dephosphorylation	0.519	0.022	0.028	0.027	0.007	0.005	0.226	0.123	0.019	6470
protein amino acid ADP-ribosylation	0.595	0.661	0.019	0.095	0.112	0.190	0.359	0.006	0.044	6471
protein amino acid methylation	0.048	0.172	0.756	0.106	0.191	0.196	0.499	0.047	0.194	6479
protein amino acid glycosylation	0.487	0.313	0.028	0.405	0.374	0.219	0.021	0.583	0.670	6486
protein amino acid N-linked glycosylation	0.815	0.019	0.026	0.651	0.285	0.743	0.810	0.032	0.632	6487
protein amino acid O-linked glycosylation	0.271	0.066	0.301	0.376	0.142	0.874	0.483	0.044	0.043	6493
GPI anchor biosynthetic process	0.985	0.187	0.198	0.280	0.656	0.742	0.379	0.975	0.161	6506
proteolysis	0.069	0.626	0.871	0.285	0.174	0.111	0.422	0.778	0.953	6508
membrane protein ectodomain proteolysis	0.008	0.302	0.298	0.165	0.230	0.104	0.834	0.716	0.176	6509
ubiquitin-dependent protein catabolic process	0.332	0.010	0.006	0.034	0.007	0.019	0.408	0.210	0.018	6511
ubiquitin cycle	0.505	0.010	0.006	0.031	0.007	0.012	0.387	0.019	0.003	6512
amino acid metabolic process	0.439	0.289	0.294	0.512	0.007	0.091	0.044	0.680	0.170	6520
glutamine metabolic process	0.701	0.284	0.295	0.149	0.638	0.316	0.255	0.047	0.019	6541
protein targeting	0.406	0.225	0.133	0.016	0.057	0.087	0.150	0.180	0.025	6605
protein import into nucleus	0.479	0.206	0.445	0.445	0.094	0.444	0.239	0.047	0.012	6606
protein export from nucleus	0.378	0.025	0.101	0.650	0.380	0.450	0.969	0.493	0.361	6611
protein targeting to mitochondrion	0.489	0.062	0.068	0.230	0.051	0.008	0.402	0.320	0.219	6626
lipid metabolic process	0.034	0.040	0.432	0.118	0.023	0.061	0.584	0.225	0.466	6629
fatty acid metabolic process	0.474	0.362	0.082	0.632	0.043	0.053	0.392	0.323	0.030	6631
fatty acid biosynthetic process	0.215	0.688	0.729	0.110	0.097	0.194	0.306	0.105	0.198	6633
fatty acid beta-oxidation	0.324	0.350	0.348	0.033	0.039	0.027	0.070	0.268	0.012	6635
acyl-CoA metabolic process	0.340	0.382	0.500	0.183	0.459	0.055	0.770	0.902	0.954	6637
phospholipid metabolic process	0.520	0.968	0.583	0.373	0.321	0.798	0.295	0.142	0.824	6644
sphingolipid metabolic process	0.956	0.358	0.840	0.185	0.076	0.034	0.545	0.255	0.748	6665
ceramide metabolic process	0.579	0.063	0.075	0.911	0.908	0.355	0.017	0.570	0.527	6672
leukotriene metabolic process	0.635	0.920	0.272	0.356	0.769	0.743	0.953	0.616	0.650	6691
steroid biosynthetic process	0.083	0.344	0.959	0.074	0.387	0.127	0.071	0.417	0.353	6694
cholesterol biosynthetic process	0.457	0.147	0.794	0.114	0.027	0.083	0.125	0.294	0.320	6695
C21-steroid hormone biosynthetic process	0.223	0.430	0.971	0.973	0.916	0.448	0.771	0.900	0.944	6700
aromatic compound metabolic process	0.018	0.060	0.031	0.659	0.091	0.106	0.088	0.768	0.425	6725
one-carbon compound metabolic process	0.018	0.501	0.775	0.049	0.482	0.569	0.143	0.632	0.545	6730
glutathione metabolic process	0.018	0.432	0.252	0.189	0.007	0.082	0.064	0.699	0.062	6749
ATP biosynthetic process	0.602	0.043	0.464	0.438	0.148	0.187	0.042	0.211	0.389	6754
heme biosynthetic process	0.313	0.730	0.391	0.161	0.007	0.046	0.275	0.626	0.546	6783
sulfur metabolic process	0.497	0.833	0.619	0.301	0.840	0.518	0.619	0.623	0.684	6790
phosphate metabolic process	0.917	0.196	0.113	0.046	0.760	0.182	0.254	0.571	0.606	6796
superoxide metabolic process	0.139	0.373	0.460	0.074	0.039	0.838	0.424	0.278	0.366	6801
xenobiotic metabolic process	0.066	0.670	0.425	0.436	0.867	0.146	0.285	0.717	0.868	6805
nitrogen compound metabolic process	0.759	0.031	0.078	0.715	0.083	0.181	0.771	0.083	0.096	6807
nitric oxide biosynthetic process	0.622	0.347	0.153	0.784	0.082	0.541	0.901	0.372	0.870	6809
transport	0.085	0.004	0.004	0.006	0.007	0.004	0.307	0.410	0.111	6810
ion transport	0.020	0.383	0.233	0.983	0.788	0.953	0.807	0.471	0.905	6811
cation transport	0.117	0.762	0.090	0.903	0.492	0.579	0.577	0.706	0.194	6812
potassium ion transport	0.562	0.658	0.187	0.960	0.535	0.452	0.579	0.233	0.803	6813
sodium ion transport	0.082	0.502	0.228	0.537	0.569	0.965	0.622	0.731	0.705	6814
calcium ion transport	0.057	0.147	0.031	0.955	0.116	0.655	0.683	0.612	0.909	6816
phosphate transport	0.970	0.366	0.787	0.250	0.136	0.384	0.419	0.820	0.808	6817
hydrogen transport	0.923	0.468	0.254	0.741	0.813	0.470	0.289	0.619	0.270	6818
anion transport	0.262	0.177	0.120	0.537	0.098	0.233	0.689	0.148	0.175	6820
chloride transport	0.680	0.810	0.319	0.513	0.543	0.429	1.000	0.859	0.647	6821
iron ion transport	0.284	0.577	0.474	0.008	0.112	0.023	0.055	0.040	0.128	6826
zinc ion transport	0.651	0.599	0.398	0.098	0.586	0.659	0.672	0.458	0.257	6829
water transport	0.541	0.944	0.944	0.800	0.417	0.576	0.779	0.200	0.479	6833
neurotransmitter transport	0.855	0.147	0.853	0.661	0.826	0.916	0.930	0.186	0.922	6836
mitochondrial transport	0.311	0.137	0.064	0.042	0.491	0.111	0.087	0.650	0.333	6839
amino acid transport	0.762	0.185	0.117	0.431	0.899	0.951	0.901	0.237	0.801	6865
lipid transport	0.006	0.042	0.025	0.095	0.023	0.021	0.730	0.251	0.493	6869
calcium ion homeostasis	0.030	0.553	0.737	0.679	0.539	0.408	0.104	0.615	0.710	6874
iron ion homeostasis	0.757	0.779	0.516	0.008	0.073	0.015	0.006	0.019	0.357	6879
regulation of pH	0.436	0.197	0.136	0.057	0.537	0.990	0.558	0.546	0.466	6885
intracellular protein transport	0.452	0.010	0.005	0.151	0.037	0.019	0.382	0.134	0.011	6886
exocytosis	0.136	0.029	0.016	0.412	0.232	0.420	0.742	0.301	0.322	6887
ER to Golgi vesicle-mediated transport	0.311	0.010	0.006	0.014	0.020	0.044	0.508	0.386	0.056	6888
intra-Golgi vesicle-mediated transport	0.667	0.062	0.087	0.270	0.057	0.033	0.343	0.128	0.360	6891
endocytosis	0.587	0.026	0.046	0.155	0.163	0.005	0.222	0.027	0.002	6897
receptor-mediated endocytosis	0.891	0.789	0.183	0.603	0.632	0.163	0.068	0.024	0.463	6898
vesicle docking during exocytosis	0.618	0.018	0.068	0.500	0.678	0.533	0.575	0.801	0.779	6904
phagocytosis	0.203	0.178	0.039	0.591	0.784	0.886	0.694	0.845	0.960	6909
phagocytosis, recognition	0.140	0.155	0.424	0.346	0.055	0.179	0.021	0.018	0.088	6910
phagocytosis, engulfment	0.018	0.488	0.649	0.350	0.062	0.474	0.016	0.010	0.109	6911
nucleocytoplasmic transport	0.083	0.241	0.016	0.583	0.135	0.147	0.368	0.705	0.166	6913
autophagy	0.803	0.048	0.054	0.587	0.195	0.024	0.412	0.069	0.029	6914
apoptosis	0.015	0.022	0.004	0.008	0.048	0.005	0.499	0.077	0.079	6915
anti-apoptosis	0.095	0.002	0.016	0.008	0.039	0.015	0.281	0.063	0.036	6916
induction of apoptosis	0.139	0.455	0.063	0.043	0.259	0.395	0.229	0.149	0.127	6917
caspase activation	0.662	0.485	0.278	0.145	0.063	0.072	0.344	0.073	0.431	6919
cell motility	0.007	0.044	0.022	0.008	0.007	0.013	0.139	0.110	0.030	6928
chemotaxis	0.158	0.900	0.999	0.789	0.992	0.906	0.531	0.298	0.629	6935
muscle contraction	0.451	0.191	0.660	0.036	0.012	0.079	0.296	0.885	0.293	6936
regulation of muscle contraction	0.127	0.123	0.084	0.246	0.649	0.039	0.603	0.554	0.071	6937

Supplemental Table 1. Complete list of Gene Ontology Biological Processes from the SAFE analysis

	Neocortex p-values			Heart p-values			Muscle p-values			
GO_term	Age	CR	Resv	Age	CR	Resv	Age	CR	Resv	GO_ID_SAFE
smooth muscle contraction	0.386	0.351	0.985	0.079	0.472	0.068	0.144	0.620	0.588	6939
striated muscle contraction	0.780	0.943	0.938	0.460	0.056	0.307	0.083	0.832	0.135	6941
membrane fusion	0.760	0.134	0.006	0.932	0.357	0.033	0.254	0.193	0.561	6944
response to stress	0.443	0.140	0.340	0.008	0.033	0.040	0.143	0.373	0.213	6950
defense response	0.948	0.991	0.992	0.999	0.990	0.948	0.953	0.962	0.970	6952
acute-phase response	0.785	0.898	0.863	0.684	0.746	0.764	0.823	0.968	0.669	6953
inflammatory response	0.047	0.986	0.994	0.693	0.825	0.845	0.248	0.304	0.285	6954
immune response	0.226	0.943	0.995	1.000	0.972	0.967	0.234	0.461	0.778	6955
complement activation	0.120	0.314	0.871	0.926	0.523	0.324	0.306	0.769	0.585	6956
complement activation, alternative pathway	0.788	0.579	0.935	0.461	0.596	0.814	0.471	0.916	0.506	6957
complement activation, classical pathway	0.356	0.659	0.997	0.919	0.697	0.292	0.075	0.795	0.769	6958
humoral immune response	0.537	0.796	0.882	0.946	0.921	0.811	0.234	0.953	0.708	6959
cellular defense response	0.525	0.967	0.972	0.565	0.520	0.760	0.820	0.850	0.836	6968
response to DNA damage stimulus	0.605	0.464	0.194	0.008	0.020	0.038	0.002	0.312	0.303	6974
response to oxidative stress	0.661	0.076	0.166	0.008	0.007	0.094	0.034	0.238	0.340	6979
response to unfolded protein	0.593	0.025	0.209	0.017	0.049	0.013	0.130	0.039	0.684	6986
chromosome organization and biogenesis (sensu Eukaryota)	0.569	0.450	0.322	0.118	0.246	0.350	0.756	0.187	0.234	7001
telomere maintenance via telomerase	0.104	0.534	0.696	0.066	0.375	0.597	0.364	0.842	0.430	7004
cytoskeleton organization and biogenesis	0.695	0.588	0.962	0.452	0.013	0.038	0.437	0.781	0.195	7010
actin filament organization	0.085	0.018	0.042	0.030	0.184	0.435	0.293	0.016	0.015	7015
microtubule-based process	0.138	0.052	0.006	0.068	0.033	0.143	0.035	0.589	0.777	7017
microtubule-based movement	0.451	0.089	0.006	0.491	0.653	0.901	0.579	0.605	0.900	7018
peroxisome organization and biogenesis	0.138	0.010	0.006	0.152	0.026	0.015	0.531	0.871	0.014	7031
lysosome organization and biogenesis	0.076	0.157	0.062	0.245	0.649	0.314	0.083	0.570	0.116	7040
ribosome biogenesis	0.845	0.010	0.110	0.160	0.051	0.025	0.020	0.594	0.243	7046
cell cycle	0.499	0.022	0.005	0.124	0.007	0.017	0.272	0.456	0.058	7049
cell cycle arrest	0.899	0.110	0.058	0.028	0.039	0.046	0.196	0.236	0.157	7050
chromosome segregation	0.099	0.539	0.084	0.028	0.288	0.197	0.342	0.230	0.353	7059
mitosis	0.269	0.113	0.006	0.056	0.013	0.005	0.243	0.657	0.400	7067
mitotic chromosome condensation	0.402	0.097	0.208	0.839	0.392	0.032	0.433	0.970	0.941	7076
meiosis	0.716	0.592	0.681	0.783	0.412	0.977	0.347	0.873	0.987	7126
meiotic recombination	0.436	0.894	0.788	0.454	0.615	0.398	0.271	0.683	0.843	7131
cell communication	0.267	0.017	0.102	0.096	0.461	0.176	0.327	0.508	0.201	7154
cell adhesion	0.005	0.064	0.024	0.288	0.067	0.503	0.055	0.440	0.661	7155
homophilic cell adhesion	0.131	0.695	0.274	0.781	0.466	0.630	0.371	0.889	1.000	7156
heterophilic cell adhesion	0.254	0.047	0.026	0.674	0.393	0.777	0.018	0.360	0.638	7157
cell-matrix adhesion	0.045	0.587	0.615	0.027	0.033	0.550	0.126	0.325	0.755	7160
negative regulation of cell adhesion	0.129	0.233	0.395	0.746	0.152	0.057	0.249	0.629	0.686	7162
establishment and/or maintenance of cell polarity	0.600	0.060	0.095	0.038	0.287	0.039	0.381	0.023	0.265	7163
signal transduction	0.145	0.680	0.949	0.767	0.491	0.304	0.044	0.012	0.803	7165
cell surface receptor linked signal transduction	0.658	0.951	0.975	0.992	0.845	0.723	0.558	0.380	0.712	7166
transmembrane receptor protein tyrosine kinase signaling pathw	0.301	0.752	0.846	0.532	0.755	0.463	0.486	0.275	0.682	7169
epidermal growth factor receptor signaling pathway	0.140	0.262	0.125	0.214	0.056	0.128	0.793	0.070	0.114	7173
transmembrane receptor protein serine/threonine kinase signalir	0.577	0.707	0.587	0.180	0.088	0.050	0.145	0.015	0.160	7178
transforming growth factor beta receptor signaling pathway	0.422	0.549	0.539	0.516	0.504	0.361	0.206	0.111	0.449	7179
transmembrane receptor protein tyrosine phosphatase signaling	0.136	0.660	0.773	0.482	0.299	0.727	0.797	0.442	0.464	7185
G-protein coupled receptor protein signaling pathway	0.128	0.980	1.000	0.980	0.999	0.998	0.555	0.379	0.962	7186
G-protein signaling, coupled to cyclic nucleotide second messen	0.885	0.784	0.337	0.834	0.885	0.752	0.217	0.370	0.790	7187
G-protein signaling, coupled to cAMP nucleotide second messer	0.278	0.169	0.987	0.197	0.155	0.761	0.567	0.627	0.884	7188
G-protein signaling, adenylate cyclase activating pathway	0.533	0.413	0.436	0.864	0.649	0.343	0.040	0.413	0.118	7189
adenylate cyclase activation	0.803	0.334	0.935	0.205	0.937	0.908	0.192	0.121	0.071	7190
G-protein signaling, adenylate cyclase inhibiting pathway	0.313	0.193	0.006	0.548	0.026	0.367	0.080	0.216	0.979	7193
G-protein signaling, coupled to IP3 second messenger (phospho	0.121	0.883	0.968	0.610	0.382	0.987	0.717	0.522	0.396	7200
phospholipase C activation	0.742	0.432	0.753	0.125	0.229	0.289	0.696	0.170	0.614	7202
elevation of cytosolic calcium ion concentration	0.398	0.514	0.997	0.941	0.755	0.989	0.418	0.707	0.862	7204
protein kinase C activation	0.399	0.019	0.233	0.534	0.136	0.308	0.546	0.850	0.751	7205
gamma-aminobutyric acid signaling pathway	0.373	0.676	0.066	0.949	0.577	0.621	0.932	0.443	0.158	7214
glutamate signaling pathway	0.419	0.088	0.064	0.924	0.287	0.528	0.351	0.245	0.594	7215
neuropeptide signaling pathway	0.051	0.357	0.672	0.743	0.708	0.912	0.445	0.407	0.996	7218
Notch signaling pathway	0.443	0.035	0.278	0.058	0.129	0.013	0.132	0.006	0.024	7219
frizzled signaling pathway	0.924	0.054	0.118	0.288	0.069	0.487	0.006	0.054	0.009	7222
Wnt receptor signaling pathway, calcium modulating pathway	0.817	0.812	0.978	0.772	0.695	0.801	0.811	0.845	0.972	7223
smoothened signaling pathway	0.376	0.700	0.763	0.379	0.131	0.379	0.769	0.119	0.490	7224
integrin-mediated signaling pathway	0.961	0.377	0.780	0.177	0.282	0.532	0.479	0.781	0.999	7229
intracellular signaling cascade	0.096	0.005	0.077	0.001	0.046	0.067	0.267	0.089	0.101	7242
protein kinase cascade	0.226	0.010	0.062	0.053	0.147	0.067	0.158	0.714	0.122	7243
I-kappaB kinase/NF-kappaB cascade	0.178	0.934	0.952	0.618	0.890	0.330	0.832	0.065	0.226	7249
JNK cascade	0.154	0.010	0.437	0.524	0.110	0.047	0.481	0.218	0.655	7254
activation of JNK activity	0.099	0.325	0.853	0.345	0.022	0.474	0.646	0.138	0.248	7257
JAK-STAT cascade	0.548	0.342	0.179	0.054	0.221	0.293	0.851	0.300	0.031	7259
small GTPase mediated signal transduction	0.034	0.004	0.002	0.002	0.007	0.005	0.614	0.258	0.058	7264
Ras protein signal transduction	0.498	0.265	0.092	0.224	0.161	0.030	0.106	0.328	0.328	7265
Rho protein signal transduction	0.202	0.018	0.016	0.008	0.024	0.005	0.761	0.426	0.067	7266
cell-cell signaling	0.332	0.822	0.997	0.903	0.962	0.653	0.343	0.161	0.997	7267
synaptic transmission	0.164	0.052	0.028	0.956	0.956	0.862	0.478	0.640	0.911	7268
neurotransmitter secretion	0.297	0.202	0.006	0.908	0.807	0.881	0.209	0.029	0.073	7269
synaptic transmission, cholinergic	0.376	0.766	0.823	0.086	0.903	0.202	0.633	0.361	0.404	7271
multicellular organismal development	0.504	0.275	0.996	0.442	0.837	0.586	0.102	0.289	0.739	7275
gamete generation	0.063	0.263	0.675	0.530	0.044	0.784	0.101	0.104	0.683	7276
germ cell development	0.235	0.010	0.287	0.649	0.131	0.272	0.819	0.915	0.711	7281
spermatogenesis	0.688	0.956	0.927	0.959	0.865	0.632	0.752	0.637	0.938	7283
spermatid development	0.752	0.109	0.619	0.387	0.510	0.354	0.124	0.213	0.003	7286
female gamete generation	0.967	0.946	0.890	0.383	0.820	0.409	0.050	0.436	0.998	7292
single fertilization	0.994	0.748	0.258	0.125	0.683	0.440	0.569	0.678	0.676	7338
binding of sperm to zona pellucida	0.778	0.764	0.713	1.000	0.877	0.979	0.772	0.480	0.944	7339
acrosome reaction	0.516	0.600	0.829	0.337	0.400	0.449	0.791	0.067	0.803	7340
determination of left/right symmetry	0.496	0.558	0.987	0.217	0.159	0.537	0.300	0.141	0.407	7368
pattern specification process	0.928	0.971	0.958	0.964	0.558	0.925	0.745	0.459	0.520	7389
ectoderm development	0.338	0.517	0.591	0.742	0.776	0.480	0.466	0.831	0.972	7398
nervous system development	0.058	0.010	0.005	0.114	0.941	0.665	0.021	0.412	0.956	7399
axonogenesis	0.045	0.144	0.221	0.157	0.286	0.058	0.483	0.211	0.998	7409
axon guidance	0.023	0.367	0.209	0.885	0.737	0.857	0.060	0.555	0.953	7411
synaptogenesis	0.643	0.197	0.051	0.073	0.102	0.138	0.108	0.025	0.245	7416
central nervous system development	0.267	0.440	0.616	0.433	0.448	0.929	0.765	0.511	0.557	7417
brain development	0.023	0.056	0.009	0.122	0.218	0.048	0.144	0.834	0.961	7420
peripheral nervous system development	0.129	0.154	0.882	0.279	0.864	0.241	0.994	0.985	0.695	7422
sensory organ development	0.740	0.589	0.255	0.962	0.928	0.979	0.539	0.929	0.813	7423
endoderm development	0.427	0.594	0.302	0.733	0.406	0.132	0.037	0.153	0.031	7492

Supplemental Table 1. Complete list of Gene Ontology Biological Processes from the SAFE analysis										
	Neocortex p-values			Heart p-values			Muscle p-values			
GO_term	Age	CR	Resv	Age	CR	Resv	Age	CR	Resv	GO_ID_SAFE
mesoderm development	0.818	0.649	0.666	0.167	0.201	0.070	0.872	0.442	0.891	7498
heart development	0.555	0.096	0.274	0.218	0.007	0.017	0.317	0.138	0.479	7507
muscle development	0.943	0.960	0.996	0.056	0.007	0.169	0.377	0.511	0.089	7517
striated muscle development	0.994	0.256	0.768	0.059	0.087	0.502	0.227	0.121	0.039	7519
sex differentiation	0.978	0.929	0.980	0.523	0.883	0.867	0.818	0.362	0.994	7548
pregnancy	0.712	0.851	0.988	0.392	0.283	0.546	0.549	0.225	0.037	7565
embryo implantation	0.124	0.734	0.316	0.715	0.176	0.460	0.888	0.345	0.826	7566
cell aging	0.167	0.236	0.058	0.554	0.730	0.672	0.763	0.666	0.898	7569
physiological process	0.436	0.469	0.201	0.754	0.123	0.370	0.959	0.884	0.750	7582
response to nutrient	0.015	0.061	0.190	0.068	0.058	0.319	0.129	0.165	0.454	7584
respiratory gaseous exchange	0.421	0.993	0.989	0.211	0.465	0.199	0.703	0.117	0.476	7585
digestion	0.394	0.775	0.946	0.950	0.597	0.848	0.289	0.910	0.913	7586
excretion	0.787	0.913	0.991	0.789	0.180	0.253	0.278	0.191	0.927	7588
lactation	0.488	0.025	0.232	0.162	0.919	0.532	0.094	0.657	0.308	7595
blood coagulation	0.057	0.603	0.807	0.784	0.284	0.187	0.310	0.298	0.406	7596
sensory perception	0.238	0.027	0.476	0.958	0.915	0.930	0.168	0.482	0.340	7600
visual perception	0.941	0.346	0.265	0.923	0.880	0.816	0.097	0.887	0.984	7601
phototransduction	0.898	0.759	0.355	0.291	0.768	0.807	0.146	0.946	0.766	7602
sensory perception of sound	0.383	0.343	0.649	0.829	0.620	0.963	0.847	0.273	0.388	7605
sensory perception of chemical stimulus	0.947	0.887	0.990	0.872	0.821	0.706	0.204	0.031	0.075	7606
sensory perception of smell	0.709	0.983	1.000	0.851	0.664	0.976	0.600	0.897	0.926	7608
behavior	0.175	0.178	0.272	0.053	0.663	0.657	0.077	0.345	0.886	7610
learning and/or memory	0.036	0.269	0.388	0.772	0.494	0.368	0.300	0.379	0.703	7611
learning	0.170	0.164	0.171	0.943	0.669	0.301	0.071	0.006	0.361	7612
memory	0.205	0.101	0.021	0.019	0.020	0.012	0.800	0.086	0.881	7613
circadian rhythm	0.064	0.708	0.083	0.193	0.303	0.734	0.791	0.573	0.952	7623
locomotory behavior	0.064	0.128	0.006	0.718	0.620	0.789	0.635	0.825	0.917	7626
adult walking behavior	0.474	0.757	0.077	0.195	0.242	0.443	0.155	0.488	0.897	7628
feeding behavior	0.072	0.786	0.962	0.713	0.420	0.960	0.387	0.908	0.922	7631
circulation	0.008	0.420	0.729	0.608	0.072	0.082	0.014	0.099	0.044	8015
regulation of heart contraction	0.365	0.036	0.043	0.436	0.256	0.060	0.093	0.816	0.908	8016
tRNA processing	0.980	0.267	0.513	0.008	0.558	0.090	0.867	0.259	0.014	8033
protein localization	0.075	0.104	0.230	0.156	0.669	0.456	0.489	0.017	0.076	8104
biological_process	0.004	0.008	0.001	0.034	0.023	0.003	0.299	0.528	0.067	8150
metabolic process	0.023	0.269	0.013	0.035	0.033	0.012	0.101	0.936	0.033	8152
steroid metabolic process	0.069	0.061	0.323	0.098	0.135	0.533	0.414	0.274	0.137	8202
cholesterol metabolic process	0.008	0.021	0.084	0.117	0.053	0.042	0.928	0.285	0.149	8203
blood pressure regulation	0.599	0.968	0.124	0.156	0.214	0.264	0.445	0.718	0.665	8217
cell death	0.036	0.769	0.612	0.187	0.166	0.047	0.772	0.650	0.416	8219
sulfate transport	0.447	0.637	0.897	0.244	0.532	0.934	0.303	0.489	0.408	8272
regulation of G-protein coupled receptor protein signaling pathwa	0.242	0.552	0.353	0.940	0.244	0.055	0.584	0.142	0.874	8277
cell proliferation	0.321	0.007	0.766	0.018	0.086	0.010	0.318	0.173	0.280	8283
positive regulation of cell proliferation	0.303	0.139	0.861	0.110	0.164	0.096	0.325	0.084	0.194	8284
negative regulation of cell proliferation	0.045	0.063	0.556	0.214	0.222	0.080	0.055	0.095	0.090	8285
insulin receptor signaling pathway	0.130	0.179	0.017	0.085	0.654	0.216	0.039	0.279	0.054	8286
isoprenoid biosynthetic process	0.852	0.406	0.222	0.286	0.052	0.433	0.310	0.417	0.070	8299
adult locomotory behavior	0.934	0.262	0.136	0.414	0.578	0.097	0.006	0.267	0.258	8344
regulation of cell shape	0.043	0.010	0.126	0.026	0.033	0.018	0.251	0.006	0.120	8360
RNA splicing	0.694	0.010	0.012	0.018	0.017	0.005	0.346	0.070	0.016	8380
fibroblast growth factor receptor signaling pathway	0.139	0.195	0.389	0.928	0.410	0.196	0.090	0.166	0.682	8543
epidermis development	0.879	0.968	0.960	0.963	0.679	0.822	0.683	0.995	0.978	8544
male gonad development	0.844	0.919	0.972	0.771	0.833	0.752	0.402	0.282	0.812	8584
lipid biosynthetic process	0.157	0.194	0.672	0.024	0.003	0.035	0.057	0.471	0.291	8610
induction of apoptosis by extracellular signals	0.078	0.410	0.578	0.008	0.251	0.857	0.455	0.297	0.878	8624
induction of apoptosis via death domain receptors	0.972	0.419	0.535	0.793	0.429	0.540	0.909	0.292	0.940	8625
induction of apoptosis by intracellular signals	0.573	0.105	0.115	0.155	0.410	0.769	0.781	0.127	0.150	8629
apoptotic program	0.404	0.342	0.243	0.219	0.445	0.248	0.374	0.030	0.033	8632
carbohydrate transport	0.513	0.567	0.341	0.656	0.721	0.627	0.273	0.945	0.771	8643
amino acid biosynthetic process	0.287	0.036	0.030	0.492	0.337	0.449	0.748	0.690	0.622	8652
phospholipid biosynthetic process	0.176	0.537	0.069	0.203	0.101	0.232	0.573	0.838	0.224	8654
biosynthetic process	0.931	0.040	0.100	0.224	0.015	0.125	0.560	0.278	0.178	9058
aromatic amino acid family metabolic process	0.845	0.545	0.981	0.660	0.395	0.468	0.466	0.366	0.849	9072
nucleoside metabolic process	0.815	0.366	0.575	0.381	0.341	0.936	0.233	0.426	0.492	9116
nucleotide metabolic process	0.939	0.172	0.039	0.063	0.051	0.052	0.157	0.368	0.114	9117
nucleotide biosynthetic process	0.390	0.656	0.250	0.292	0.689	0.393	0.295	0.949	0.847	9165
cyclic nucleotide biosynthetic process	0.429	0.466	0.660	0.079	0.162	0.248	0.710	0.209	0.341	9190
protein secretion	0.324	0.899	0.851	0.062	0.103	0.015	0.705	0.566	0.571	9306
oligosaccharide biosynthetic process	0.127	0.296	0.030	0.401	0.727	0.648	0.094	0.342	0.495	9312
response to radiation	0.815	0.234	0.962	0.162	0.374	0.205	0.020	0.696	0.284	9314
phosphoenolpyruvate-dependent sugar phosphotransferase sys	0.331	0.985	0.434	0.266	0.036	0.293	0.380	0.676	0.243	9401
response to heat	0.710	0.227	0.672	0.364	0.110	0.095	0.560	0.068	0.662	9408
response to cold	0.662	0.107	0.256	0.248	0.036	0.191	0.576	0.760	0.790	9409
response to UV	0.107	0.143	0.642	0.261	0.812	0.205	0.689	0.112	0.181	9411
response to biotic stimulus	0.068	0.448	0.979	0.401	1.000	0.672	0.729	0.821	0.075	9607
response to wounding	0.134	0.116	0.802	0.639	0.095	0.415	0.336	0.292	0.788	9611
response to virus	0.888	0.958	0.669	0.935	0.908	0.863	0.934	0.919	0.788	9615
response to toxin	0.123	0.305	0.823	0.135	0.178	0.015	0.523	0.421	0.556	9636
anatomical structure morphogenesis	0.305	0.437	0.637	0.502	0.503	0.326	0.538	0.564	0.973	9653
hormone-mediated signaling	0.832	0.590	0.852	0.571	0.421	0.150	0.726	0.309	0.552	9755
embryonic development	0.770	0.606	0.569	0.043	0.205	0.094	0.592	0.131	0.071	9790
organ morphogenesis	0.714	0.553	0.853	0.900	0.063	0.273	0.702	0.336	0.948	9887
tissue development	0.880	0.135	0.983	0.643	0.451	0.201	0.879	0.218	0.358	9888
anterior/posterior pattern formation	0.496	0.729	0.113	0.295	0.219	0.408	0.631	0.429	0.336	9952
dorsal/ventral pattern formation	0.503	0.089	0.968	0.664	0.577	0.342	0.711	0.052	0.963	9953
regulation of signal transduction	0.281	0.787	0.353	0.307	0.146	0.146	0.978	0.020	0.042	9966
negative regulation of signal transduction	0.219	0.597	0.328	0.339	0.182	0.446	0.361	0.253	0.421	9968
glial cell differentiation	0.274	0.205	0.884	0.870	0.503	0.376	0.145	0.526	0.449	10001
gastrulation (sensu Mammalia)	0.547	0.081	0.037	0.374	0.063	0.022	0.078	0.455	0.824	10003
protein transport	0.100	0.008	0.005	0.008	0.007	0.005	0.350	0.060	0.016	15031
DNA integration	0.333	0.510	0.830	0.414	0.140	0.290	0.868	0.199	0.184	15074
oxygen transport	0.978	0.068	0.140	0.654	0.830	0.945	0.342	0.371	0.070	15671
organic anion transport	0.874	0.972	0.772	0.699	0.640	0.911	0.378	0.930	0.381	15711
glucose transport	0.226	0.317	0.361	0.019	0.761	0.192	0.379	0.620	0.717	15758
ATP synthesis coupled proton transport	0.791	0.059	0.607	0.618	0.467	0.286	0.015	0.297	0.206	15986
proton transport	0.781	0.116	0.287	0.656	0.687	0.298	0.129	0.547	0.620	15992
lipid catabolic process	0.148	0.748	0.303	0.591	0.076	0.475	0.859	0.507	0.517	16042
cellular component organization and biogenesis	0.644	0.088	0.222	0.130	0.115	0.094	0.294	0.352	0.584	16043
membrane organization and biogenesis	0.985	0.970	0.806	0.976	0.181	0.158	0.731	0.019	0.261	16044

Supplemental Table 1. Complete list of Gene Ontology Biological Processes from the SAFE analysis

	Neocortex p-values			Heart p-values			Muscle p-values			
GO_term	Age	CR	Resv	Age	CR	Resv	Age	CR	Resv	GO_ID_SAFE
cell growth	0.615	0.655	0.618	0.035	0.016	0.023	0.800	0.166	0.347	16049
carbohydrate biosynthetic process	0.305	0.260	0.550	0.144	0.647	0.290	0.923	0.006	0.520	16051
Wnt receptor signaling pathway	0.959	0.016	0.198	0.418	0.157	0.398	0.472	0.336	0.453	16055
immunoglobulin mediated immune response	0.107	0.911	0.960	0.492	0.561	0.877	0.736	0.241	0.105	16064
mRNA metabolic process	0.775	0.055	0.581	0.162	0.016	0.026	0.398	0.212	0.036	16071
sterol biosynthetic process	0.169	0.356	0.866	0.014	0.044	0.248	0.060	0.088	0.292	16126
snRNA processing	0.089	0.755	0.244	0.771	0.166	0.951	0.443	0.511	0.151	16180
vesicle-mediated transport	0.122	0.018	0.006	0.074	0.186	0.083	0.040	0.272	0.125	16192
endosome transport	0.223	0.658	0.631	0.068	0.235	0.085	0.481	0.097	0.203	16197
phosphorylation	0.153	0.188	0.006	0.382	0.257	0.429	0.020	0.084	0.626	16310
morphogenesis of embryonic epithelium	0.189	0.132	0.150	0.481	0.226	0.752	0.211	0.705	0.963	16331
cell-cell adhesion	0.029	0.215	0.157	0.188	0.542	0.371	0.146	0.523	0.520	16337
calcium-independent cell-cell adhesion	0.497	0.950	0.989	0.753	0.992	0.923	0.673	0.633	0.718	16338
calcium-dependent cell-cell adhesion	0.822	0.856	0.225	0.853	0.554	0.269	0.865	0.380	0.900	16339
dendrite development	0.060	0.774	0.855	0.280	0.630	0.560	0.042	0.274	0.519	16358
cell migration	0.403	0.202	0.025	0.064	0.007	0.005	0.412	0.538	0.409	16477
negative regulation of transcription	0.040	0.338	0.048	0.081	0.074	0.032	0.753	0.622	0.355	16481
protein processing	0.119	0.229	0.045	0.733	0.671	0.368	0.043	0.138	0.115	16485
negative regulation of angiogenesis	0.813	0.461	0.419	0.281	0.210	0.524	0.807	0.263	0.044	16525
protein ubiquitination	0.368	0.101	0.731	0.305	0.015	0.046	0.212	0.358	0.512	16567
chromatin modification	0.635	0.023	0.005	0.060	0.028	0.015	0.343	0.281	0.012	16568
histone methylation	0.488	0.137	0.114	0.943	0.230	0.385	0.654	0.190	0.029	16571
cell wall catabolic process	0.803	0.680	0.738	0.123	0.444	0.130	0.391	0.954	0.863	16998
peptidyl-serine phosphorylation	0.104	0.427	0.310	0.656	0.509	0.192	0.968	0.314	0.198	18105
peptidyl-tyrosine phosphorylation	0.061	0.165	0.780	0.086	0.106	0.253	0.212	0.254	0.298	18108
peptide cross-linking	0.122	0.852	0.906	0.592	0.953	0.715	0.042	0.540	0.903	18149
protein amino acid N-linked glycosylation via asparagine	0.172	0.238	0.006	0.008	0.188	0.107	0.840	0.379	0.131	18279
protein-chromophore linkage	0.180	0.414	0.511	0.395	0.752	0.965	0.261	0.847	0.474	18298
protein amino acid prenylation	0.475	0.420	0.274	0.292	0.236	0.239	0.542	0.861	0.666	18346
cytokine and chemokine mediated signaling pathway	0.840	0.182	0.955	0.128	0.036	0.147	0.279	0.189	0.302	19221
sensory perception of pain	0.071	0.032	0.187	0.353	0.111	0.358	0.618	0.814	0.739	19233
response to pheromone	0.863	0.914	0.992	0.930	0.736	0.870	0.397	0.055	0.407	19236
pyridine nucleotide biosynthetic process	0.858	0.599	0.456	0.691	0.141	0.434	0.936	0.488	0.771	19363
leukotriene biosynthetic process	0.664	0.748	0.636	0.087	0.608	0.157	0.969	0.465	0.265	19370
protein metabolic process	0.299	0.042	0.006	0.718	0.045	0.065	0.367	0.368	0.670	19538
calcium-mediated signaling	0.198	0.988	0.862	0.807	0.327	0.647	0.419	0.264	0.671	19722
antimicrobial humoral response (sensu Vertebrata)	0.106	0.909	0.787	0.513	0.888	0.988	0.077	0.441	0.903	19735
cytolysis	0.598	0.748	0.603	0.424	0.940	0.598	0.720	0.959	0.866	19835
antigen processing and presentation	0.247	0.927	0.664	0.925	0.627	0.891	0.447	0.808	0.564	19882
antigen processing and presentation of exogenous peptide antig	0.023	0.411	0.714	0.569	0.469	0.810	0.577	0.428	0.160	19886
metal ion transport	0.856	0.147	0.221	0.252	0.379	0.717	0.820	0.962	0.977	30001
lamellipodium biogenesis	0.101	0.432	0.332	0.008	0.031	0.182	0.333	0.191	0.402	30032
actin cytoskeleton organization and biogenesis	0.070	0.003	0.015	0.008	0.016	0.005	0.186	0.053	0.226	30036
actin filament polymerization	0.471	0.290	0.015	0.065	0.589	0.257	0.044	0.625	0.776	30041
actin filament-based movement	0.668	0.437	0.538	0.472	0.125	0.341	0.012	0.252	0.025	30048
insulin secretion	0.668	0.321	0.032	0.926	0.796	0.795	0.425	0.205	0.809	30073
hemopoiesis	0.311	0.825	0.460	0.212	0.007	0.445	0.296	0.368	0.207	30097
myeloid cell differentiation	0.400	0.077	0.147	0.175	0.051	0.320	0.290	0.839	0.872	30099
regulation of endocytosis	0.138	0.095	0.006	0.283	0.090	0.141	0.340	0.211	0.360	30100
cell differentiation	0.087	0.064	0.646	0.111	0.220	0.298	0.020	0.311	0.097	30154
regulation of cell adhesion	0.104	0.024	0.067	0.547	0.033	0.044	0.283	0.429	0.518	30155
protein catabolic process	0.188	0.032	0.053	0.028	0.023	0.005	0.076	0.399	0.293	30163
platelet activation	0.796	0.937	0.618	0.752	0.075	0.238	0.169	0.440	0.913	30168
negative regulation of Wnt receptor signaling pathway	0.561	0.120	0.878	0.239	0.434	0.603	0.751	0.367	0.297	30178
neuron differentiation	0.780	0.841	0.597	0.291	0.760	0.675	0.257	0.906	0.897	30182
B cell differentiation	0.676	0.946	0.627	0.655	0.405	0.259	0.643	0.337	0.470	30183
extracellular matrix organization and biogenesis	0.369	0.148	0.684	0.064	0.162	0.414	0.957	0.432	0.580	30198
collagen fibril organization	0.704	0.329	0.052	0.576	0.124	0.186	0.275	0.891	0.870	30199
keratinocyte differentiation	0.133	0.714	0.930	0.820	0.671	0.973	0.968	0.372	0.701	30216
T cell differentiation	0.217	0.225	0.056	0.936	0.732	0.729	0.046	0.926	0.803	30217
erythrocyte differentiation	0.348	0.133	0.542	0.168	0.953	0.537	0.561	0.932	0.134	30218
bone mineralization	0.143	0.787	0.618	0.924	0.822	0.671	0.192	0.021	0.398	30282
negative regulation of cell growth	0.106	0.041	0.102	0.330	0.116	0.717	0.104	0.104	0.499	30308
sperm motility	0.869	0.783	0.302	0.234	0.480	0.650	0.189	0.528	0.814	30317
melanocyte differentiation	0.669	0.152	0.054	0.901	0.433	0.553	0.742	0.006	0.162	30318
lung development	0.920	0.144	0.497	0.306	0.089	0.095	0.716	0.157	0.894	30324
embryonic limb morphogenesis	0.333	0.138	0.793	0.774	0.741	0.528	0.429	0.023	0.639	30326
regulation of cell migration	0.227	0.010	0.481	0.177	0.015	0.377	0.585	0.809	0.375	30334
positive regulation of cell migration	0.330	0.259	0.607	0.036	0.731	0.104	0.436	0.616	0.342	30335
negative regulation of cell migration	0.504	0.105	0.154	0.108	0.048	0.688	0.283	0.402	0.067	30336
ER-associated protein catabolic process	0.711	0.652	0.364	0.588	0.109	0.368	0.249	0.144	0.272	30433
BMP signaling pathway	0.049	0.332	0.101	0.235	0.228	0.049	0.092	0.029	0.032	30509
negative regulation of BMP signaling pathway	0.868	0.249	0.313	0.441	0.007	0.052	0.157	0.416	0.770	30514
estrogen receptor signaling pathway	0.575	0.091	0.615	0.028	0.126	0.005	0.016	0.718	0.923	30520
androgen receptor signaling pathway	0.668	0.020	0.062	0.099	0.059	0.005	0.224	0.359	0.255	30521
collagen catabolic process	0.219	0.819	0.968	0.950	0.734	0.689	0.180	0.719	0.954	30574
neutrophil chemotaxis	0.114	0.597	0.583	0.073	0.752	0.216	0.598	0.414	0.772	30593
epithelial cell differentiation	0.663	0.953	0.956	0.820	0.965	0.946	0.893	0.148	0.759	30855
cortical actin cytoskeleton organization and biogenesis	0.008	0.048	0.062	0.131	0.023	0.211	0.357	0.032	0.031	30866
mammary gland development	0.951	0.077	0.143	0.386	0.931	0.914	0.110	0.765	0.730	30879
positive regulation of B cell proliferation	0.135	0.887	0.861	0.558	0.680	0.857	0.171	0.114	0.169	30890
forebrain development	0.091	0.042	0.108	0.716	0.568	0.325	0.380	0.066	0.189	30900
midbrain development	0.676	0.795	0.906	0.094	0.684	0.739	0.504	0.757	0.567	30901
hindbrain development	0.392	0.701	0.655	0.345	0.781	0.622	0.461	0.727	0.390	30902
unfolded protein response	0.202	0.867	0.555	0.038	0.808	0.716	0.490	0.558	0.552	30968
hair follicle morphogenesis	0.746	0.507	0.559	0.586	0.419	0.046	0.158	0.314	0.632	31069
neurite development	0.281	0.311	0.352	0.285	0.598	0.202	0.079	0.826	0.793	31175
keratinization	0.764	0.980	0.935	1.000	0.890	0.982	0.948	0.843	0.962	31424
regulation of ARF protein signal transduction	0.194	0.054	0.103	0.104	0.152	0.662	0.791	0.221	0.071	32012
regulation of Rho protein signal transduction	0.144	0.018	0.044	0.087	0.427	0.161	0.716	0.807	0.948	35023
embryonic hindlimb morphogenesis	0.091	0.030	0.268	0.563	0.043	0.156	0.085	0.057	0.378	35116
growth	0.260	0.788	0.551	0.132	0.592	0.038	0.137	0.313	0.244	40007
regulation of body size	0.453	0.662	0.515	0.071	0.138	0.112	0.224	0.075	0.122	40014
positive regulation of body size	0.536	0.876	0.677	0.557	0.384	0.550	0.096	0.785	0.385	40018
wound healing	0.487	0.370	0.977	0.084	0.490	0.072	0.927	0.504	0.120	42060
T cell proliferation	0.422	0.351	0.721	0.488	0.885	0.806	0.625	0.704	0.929	42098
positive regulation of T cell proliferation	0.441	0.934	0.997	0.700	0.998	0.643	0.231	0.160	0.151	42102
T cell activation	0.381	0.831	0.937	0.973	0.872	0.933	0.852	0.475	0.490	42110

Supplemental Table 1. Complete list of Gene Ontology Biological Processes from the SAFE analysis

	Neocortex p-values			Heart p-values			Muscle p-values			
GO_term	Age	CR	Resv	Age	CR	Resv	Age	CR	Resv	GO_ID_SAFE
B cell activation	0.906	0.544	0.600	0.984	0.333	0.283	0.572	0.164	0.720	42113
regulation of cell proliferation	0.781	0.020	0.024	0.086	0.036	0.142	0.006	0.012	0.076	42127
negative regulation of T cell proliferation	0.328	0.912	0.581	0.157	0.273	0.630	0.032	0.517	0.722	42130
lipoprotein metabolic process	0.016	0.992	0.632	0.728	0.056	0.453	0.927	0.723	0.800	42157
ribosome biogenesis and assembly	0.398	0.087	0.796	0.078	0.527	0.689	0.017	0.378	0.179	42254
inner ear morphogenesis	0.711	0.580	0.726	0.611	0.732	0.960	0.808	0.811	0.681	42472
odontogenesis (sensu Vertebrata)	0.296	0.217	0.912	0.529	0.339	0.254	0.705	0.497	0.667	42475
auditory receptor cell differentiation	0.116	0.122	0.047	0.918	0.540	0.924	0.821	0.725	0.046	42491
response to drug	0.530	0.614	0.365	0.734	0.345	0.227	0.012	0.139	0.086	42493
myelination	0.146	0.192	0.020	0.559	0.092	0.164	0.745	0.945	0.243	42552
glucose homeostasis	0.618	0.258	0.054	0.123	0.167	0.228	0.659	0.143	0.748	42593
cholesterol homeostasis	0.109	0.207	0.254	0.289	0.034	0.010	0.134	0.693	0.321	42632
embryonic digit morphogenesis	0.317	0.182	0.727	0.058	0.193	0.295	0.312	0.138	0.599	42733
defense response to bacterium	0.437	1.000	1.000	0.909	0.890	0.912	0.767	0.724	0.988	42742
regulation of apoptosis	0.188	0.244	0.006	0.019	0.015	0.013	0.312	0.216	0.338	42981
positive regulation of apoptosis	0.034	0.400	0.852	0.008	0.024	0.042	0.148	0.209	0.036	43065
negative regulation of apoptosis	0.549	0.091	0.082	0.347	0.007	0.043	0.037	0.036	0.048	43066
regulation of GTPase activity	0.320	0.023	0.090	0.255	0.722	0.094	0.213	0.369	0.334	43087
positive regulation of I-kappaB kinase/NF-kappaB cascade	0.582	0.534	0.457	0.668	0.047	0.053	0.889	0.099	0.475	43123
pigmentation	0.749	0.306	0.197	0.866	0.455	0.326	0.885	0.013	0.100	43473
negative regulation of neuron apoptosis	0.025	0.930	0.407	0.445	0.083	0.139	0.082	0.541	0.266	43524
cellular metabolic process	0.351	0.040	0.040	0.120	0.439	0.259	0.567	0.678	0.181	44237
cellular protein metabolic process	0.453	0.132	0.074	0.149	0.163	0.603	0.006	0.027	0.075	44267
positive regulation of interferon-gamma biosynthetic process	0.896	0.850	0.907	0.529	0.920	0.826	0.123	0.505	0.397	45078
positive regulation of interleukin-2 biosynthetic process	0.394	0.182	0.285	0.682	0.909	0.837	0.683	0.376	0.046	45086
innate immune response	0.179	0.629	0.946	0.790	0.634	0.244	0.433	0.734	0.791	45087
cell fate commitment	0.256	0.695	0.695	0.324	0.979	0.920	0.825	0.276	0.743	45165
fat cell differentiation	0.092	0.123	0.110	0.017	0.606	0.935	0.427	0.728	0.107	45444
regulation of transcription	0.286	0.912	0.750	0.579	0.264	0.292	0.560	0.376	0.688	45449
bone resorption	0.702	0.266	0.579	0.638	0.641	0.389	0.182	0.219	0.634	45453
cell redox homeostasis	0.235	0.319	0.464	0.441	0.091	0.178	0.612	0.218	0.041	45454
regulation of cell differentiation	0.849	0.024	0.006	0.040	0.389	0.160	0.505	0.404	0.738	45595
negative regulation of cell differentiation	0.198	0.111	0.158	0.186	0.041	0.400	0.596	0.059	0.579	45596
regulation of neuron differentiation	0.122	0.474	0.225	0.071	0.560	0.314	0.807	0.499	0.390	45664
negative regulation of neuron differentiation	0.641	0.376	0.304	0.459	0.240	0.164	0.342	0.750	0.902	45665
negative regulation of progression through cell cycle	0.036	0.020	0.049	0.597	0.069	0.034	0.264	0.187	0.028	45786
positive regulation of protein kinase activity	0.174	0.180	0.844	0.798	0.741	0.204	0.091	0.701	0.335	45860
negative regulation of transcription, DNA-dependent	0.475	0.018	0.015	0.717	0.013	0.036	0.313	0.069	0.018	45892
positive regulation of transcription, DNA-dependent	0.008	0.045	0.015	0.237	0.039	0.025	0.496	0.156	0.046	45893
positive regulation of transcription	0.265	0.017	0.220	0.312	0.345	0.103	0.069	0.025	0.079	45941
positive regulation of transcription from RNA polymerase II prom	0.459	0.094	0.637	0.033	0.176	0.262	0.172	0.008	0.003	45944
positive regulation of translation	0.932	0.010	0.026	0.008	0.033	0.013	0.552	0.706	0.125	45946
positive regulation of JNK cascade	0.988	0.743	0.488	0.327	0.245	0.185	0.130	0.153	0.134	46330
protein amino acid autophosphorylation	0.053	0.132	0.961	0.527	0.179	0.514	0.404	0.043	0.016	46777
phospholipid dephosphorylation	0.355	0.027	0.050	0.040	0.064	0.091	0.054	0.068	0.135	46839
intracellular transport	0.067	0.021	0.026	0.513	0.099	0.393	0.780	0.014	0.003	46907
phosphoinositide-mediated signaling	0.317	0.774	0.802	0.204	0.410	0.153	0.670	0.975	0.788	48015
focal adhesion formation	0.471	0.010	0.026	0.073	0.166	0.152	0.398	0.640	0.534	48041
pigmentation during development	0.785	0.770	0.170	0.454	0.047	0.012	0.409	0.070	0.236	48066
regulation of synaptic plasticity	0.188	0.087	0.036	0.789	0.386	0.718	0.014	0.084	0.497	48167
cell development	0.236	0.178	0.595	0.406	0.250	0.092	0.768	0.884	0.636	48468
cell maturation	0.473	0.089	0.101	0.805	0.388	0.915	0.070	0.800	0.994	48469
rhythmic process	0.021	0.408	0.343	0.143	0.261	0.906	0.854	0.048	0.326	48511
blood vessel morphogenesis	0.877	0.169	0.150	0.250	0.142	0.096	0.634	0.788	0.225	48514
lymph node development	0.606	0.533	0.870	0.917	0.196	0.041	0.086	0.042	0.859	48535
thymus development	0.937	0.638	0.830	0.790	0.925	0.525	0.663	0.379	0.346	48538
morphogenesis (sensu Mammalia)	0.434	0.179	0.089	0.230	0.007	0.017	0.827	0.529	0.663	48595
neuron fate commitment	0.170	0.123	0.622	0.220	0.475	0.618	0.846	0.722	0.809	48663
neuron development	0.859	0.949	0.983	0.262	0.720	0.158	0.523	0.561	0.967	48666
branching morphogenesis of a tube	0.596	0.136	0.470	0.671	0.353	0.528	0.642	0.182	0.802	48754
positive regulation of epithelial cell proliferation	0.224	0.540	0.569	0.199	0.466	0.117	0.625	0.385	0.190	50679
positive regulation of peptidyl-tyrosine phosphorylation	0.238	0.789	0.914	0.376	0.362	0.998	0.356	0.794	0.229	50731
positive regulation of phagocytosis	0.038	0.462	0.829	0.147	0.057	0.331	0.008	0.037	0.064	50766
regulation of immune response	0.403	0.944	0.568	0.812	0.413	0.393	0.347	0.031	0.302	50776
synapse organization and biogenesis	0.938	0.141	0.318	0.770	0.618	0.329	0.091	0.790	0.609	50808
protein stabilization	0.338	0.372	0.932	0.493	0.377	0.632	0.027	0.700	0.546	50821
T cell receptor signaling pathway	0.468	0.397	0.181	0.763	0.468	0.799	0.277	0.793	0.778	50852
cellular physiological process	0.305	0.020	0.121	0.026	0.169	0.023	0.033	0.137	0.601	50875
regulation of blood vessel size	0.570	0.975	0.568	0.281	0.751	0.813	0.704	0.500	0.378	50880
regulation of balance	0.422	0.702	0.043	0.019	0.860	0.170	0.070	0.475	0.077	50885
response to stimulus	0.878	0.724	0.924	0.723	0.964	0.975	0.818	0.847	0.990	50896
barbed-end actin filament capping	0.591	0.435	0.701	0.023	0.071	0.377	0.615	0.612	0.325	51016
actin filament bundle formation	0.302	0.162	0.599	0.244	0.167	0.195	0.149	0.444	0.120	51017
mRNA transport	0.644	0.204	0.062	0.416	0.496	0.005	0.320	0.141	0.038	51028
regulation of small GTPase mediated signal transduction	0.022	0.010	0.034	0.168	0.072	0.098	0.455	0.108	0.278	51056
chaperone cofactor-dependent protein folding	0.599	0.296	0.420	0.572	0.748	0.993	0.902	0.781	0.191	51085
activation of NF-kappaB transcription factor	0.443	0.223	0.914	0.863	0.718	0.106	0.463	0.053	0.155	51092
cartilage development	0.697	0.461	0.470	0.182	0.884	0.038	0.639	0.585	0.285	51216
protein polymerization	0.908	0.809	0.154	0.121	0.690	0.045	0.358	0.730	0.966	51258
protein homooligomerization	0.872	0.724	0.808	0.412	0.051	0.102	0.103	0.324	0.295	51260
cell division	0.469	0.099	0.006	0.312	0.007	0.005	0.297	0.656	0.267	51301
