# Supplementary material for: A Low Dose of Dietary Resveratrol Partially Mimics Caloric Restriction and Retards Aging Parameters in Mice
Source: PLoS One. 2008 Jun 4;3(6):e2264. doi: 10.1371/journal.pone.0002264 (PMC2386967; doi:10.1371/journal.pone.0002264)
Supplement: Table S2 — Necropsy results from old mice fed a control, CR or resveratrol-supplemented diet (0.04 MB PDF) [file pone.0002264.s002.pdf]

Treatment	ID	Necropsy notes
Old Control	Co-01	Found dead @ 27.7 months; spleen enlarged and cystic
Old Control	Co-02	Small tumor right lung
Old Control	Co-03	Liver tumor, right lobe
Old Control	Co-04	Splenic tumor
Old Control	Co-05	No findings
Old Control	Co-06	Large liver tumor, right lobe
Old Control	Co-07	No findings
Old Control	Co-08	No findings
Old Control	Co-09	Small liver tumor, middle lobe
Old Control	Co-10	Small liver tumor, right lobe
Old Control	Co-11	No findings
Old Control	Co-12	Large liver tumor, left lobe
Old Control	Co-13	Liver tumor, left lobe
Old Control	Co-14	Large liver tumor, right lobe
Old Control	Co-15	Liver tumors, left, right, and right middle lobes
Old Control	Co-16	Liver tumors, right, and right and right middle lobes
Old Control	Co-17	Found dead @ 23.0 months; liver pale, spleen slightly enlarged
Old Control	Co-18	Liver tumor, right lobe
Old Control	Co-19	Liver tumors, right and left middle lobes
Old Control	Co-20	Liver tumors, right and left middle lobes
Old Control	Co-21	Left eye bulging lower lid, spleen 24 x 8 x 3 mm, 2 x 3 mm liver tumor left lobe
Old Control	Co-22	Liver tumors 7 x 12 x 8 mm left lobe, 3 x 4 mm middle lobe, two 2 x 2 mm right lobe, spleen slightly enlarged
Old Control	Co-23	No findings
Old Control	Co-24	No findings
Old Control	Co-25	Liver tumor 5 x 5 x 3 mm right lobe
Old Control	Co-26	Lung tumor 8 x 8 x 7 mm right upper lobe, 22 x 7 x 3 mm spleen
Old Control	Co-27	Lung tumor 2 x 2 mm right lower lobe
Old Control	Co-28	No findings
Old Control	Co-29	No findings
Old Control	Co-30	Liver tumor 10 x 10 mm left lobe
Old CR	CR-01	One small and one large tumor on right kidney; enlarged spleen
Old CR	CR-02	No findings
Old CR	CR-03	No findings
Old CR	CR-04	No findings
Old CR	CR-05	5 mm tumor, right lung, upper lobe
Old CR	CR-06	Small tumor, left kidney
Old CR	CR-07	1 mm tumor, right lower lung
Old CR	CR-08	No findings
Old CR	CR-09	No findings
Old CR	CR-10	No findings
Old CR	CR-11	Liver tumor 2 x 2 mm middle lobe
Old CR	CR-12	No findings
Old CR	CR-13	Lung tumors 2 x 2 mm left upper lobe, 1 x 1mm right lower lobe
Old CR	CR-14	No findings
Old CR	CR-15	No findings
Old CR	CR-16	No findings
Old CR	CR-17	No findings
Old CR	CR-18	Liver tumor 15 x 8 x 8 mm right lobe, 6 x 5 x 5 mm mesenteric mass
Old resveratrol	Res-01	No findings
Old resveratrol	Res-02	No findings
Old resveratrol	Res-03	Enlarged spleen
Old resveratrol	Res-04	No findings
Old resveratrol	Res-05	Many tumors: Liver, medial lobe; hard tumor connected to stomach; tumor on left kidney, proximal end; enlarged spleen.
Old resveratrol	Res-06	No findings
Old resveratrol	Res-07	Lung tumor, left middle lobe; enlarged spleen.
Old resveratrol	Res-08	No findings
Old resveratrol	Res-09	Found dead @ 29.7 months; lung tumor, lower left lobe.
Old resveratrol	Res-10	Liver tumor, right middle lobe
Old resveratrol	Res-11	Found dead @ 22.8 months; no findings
Old resveratrol	Res-12	Liver tumor, right lobe
Old resveratrol	Res-13	3 small liver tumors, left and left middle lobes
Old resveratrol	Res-14	Large spleen tumor
Old resveratrol	Res-15	Liver tumor
Old resveratrol	Res-16	2 liver tumors, left lobe
Old resveratrol	Res-17	No findings
Old resveratrol	Res-18	Large liver tumor, right lobe
Old resveratrol	Res-19	Very large splenomegaly
Old resveratrol	Res-20	No findings
Old resveratrol	Res-21	Liver completely occupied by tumors, splenomegaly with splenic tumor
Old resveratrol	Res-22	Large lung tumor, right middle lobe
