# Supplementary figures and images for: Correction: A Low Dose of Dietary Resveratrol Partially Mimics Caloric Restriction and Retards Aging Parameters in Mice
Source: PLoS One. 2008 Jun 23;3(6):10.1371/annotation/7d56e94e-3582-413d-b987-fccd0da79081. doi: 10.1371/annotation/7d56e94e-3582-413d-b987-fccd0da79081 (PMC2656377; doi:10.1371/annotation/7d56e94e-3582-413d-b987-fccd0da79081)

Supplemental Figure 1

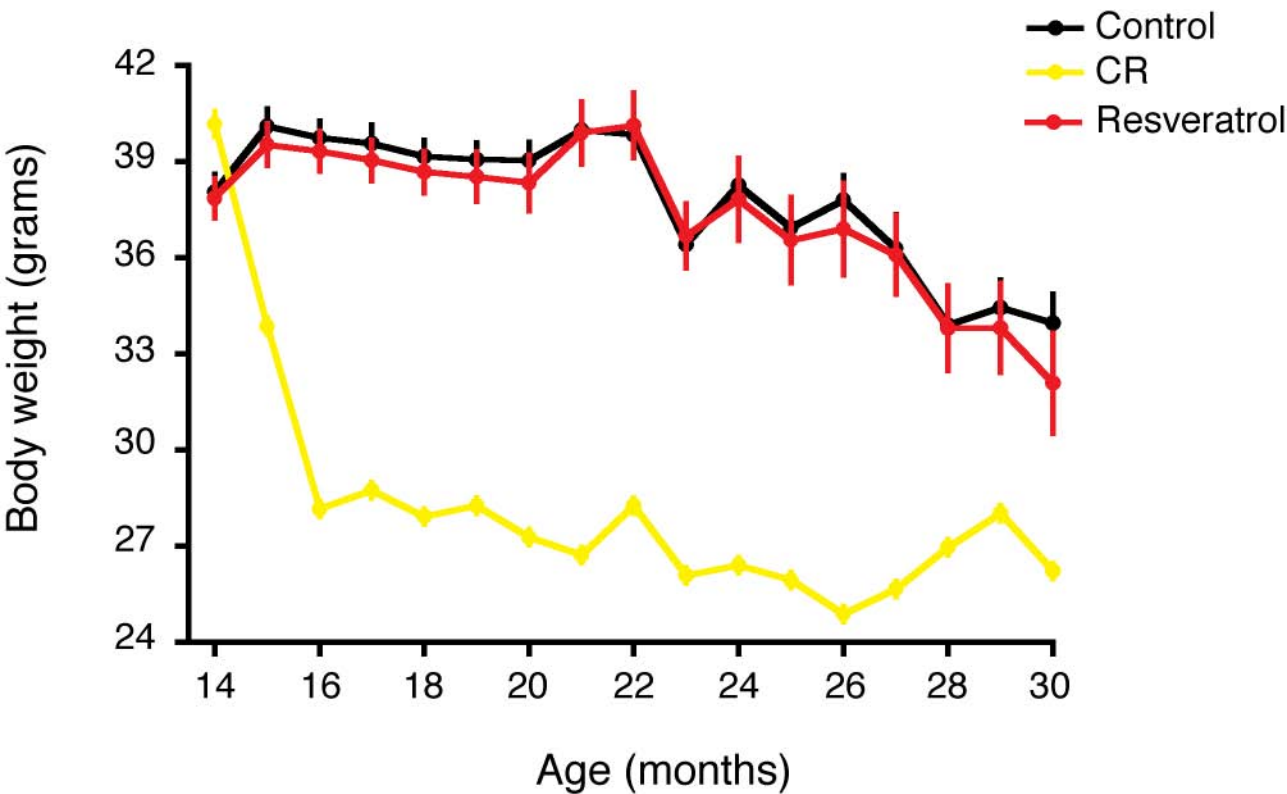

Supplement: Supplementary file 1 [file pone.7d56e94e-3582-413d-b987-fccd0da79081.s001.pdf]
