# Supplementary figures and images for: Correction: A Low Dose of Dietary Resveratrol Partially Mimics Caloric Restriction and Retards Aging Parameters in Mice
Source: PLoS One. 2008 Jun 23;3(6):10.1371/annotation/c54ef754-1962-4125-bf19-76d3ec6f19e5. doi: 10.1371/annotation/c54ef754-1962-4125-bf19-76d3ec6f19e5 (PMC2656690; doi:10.1371/annotation/c54ef754-1962-4125-bf19-76d3ec6f19e5)

## Supplemental Figure 2

### DNA Oxidation

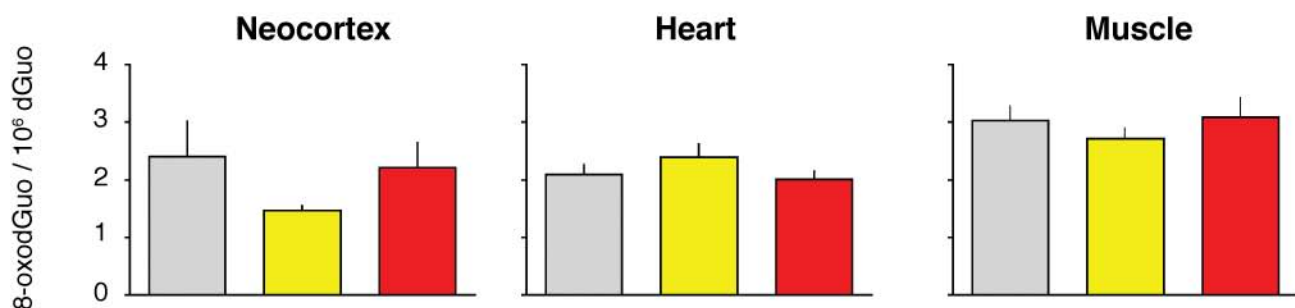

### RNA Oxidation

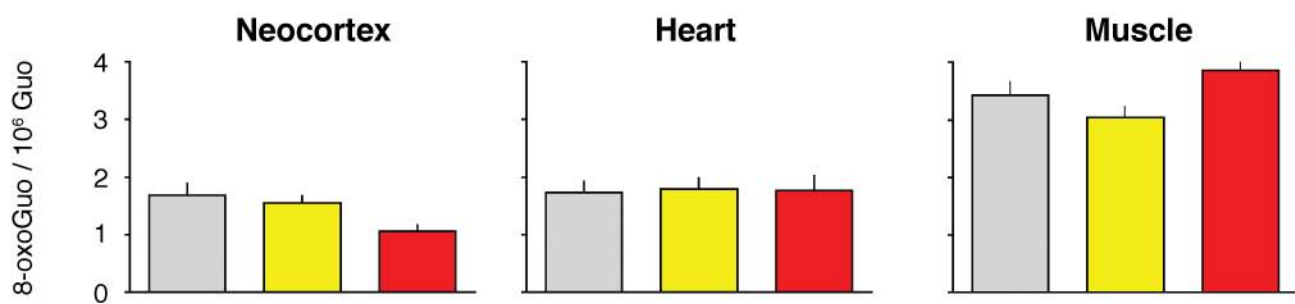

### Bound F Isoprostanes

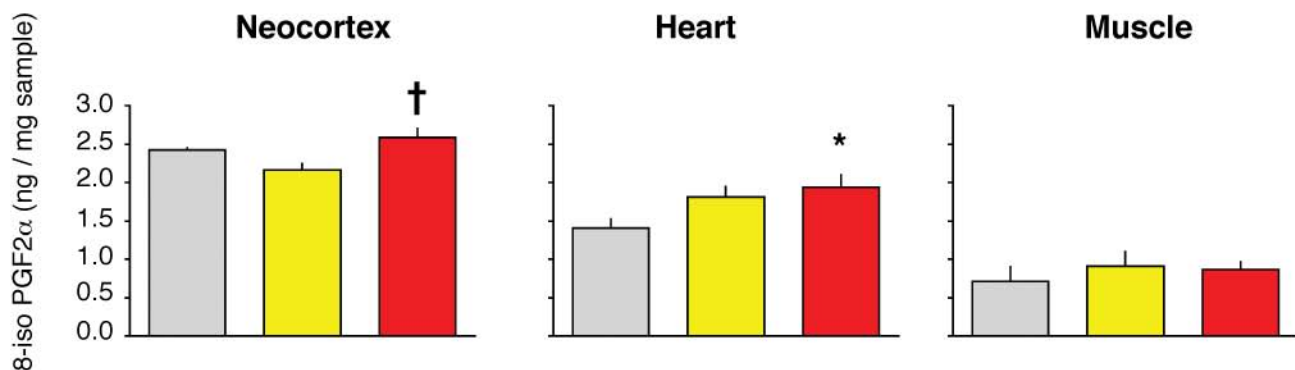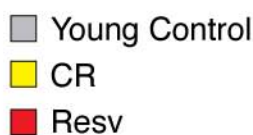

Supplement: Supplementary file 1 [file pone.c54ef754-1962-4125-bf19-76d3ec6f19e5.s001.pdf]
